# Supplementary material for: Mechanical gating of tendon fibrogenic transcription in systemic sclerosis
Source: Nat Commun. 2026 Mar 13;17:3893. doi: 10.1038/s41467-026-70395-2 (PMC13125569; doi:10.1038/s41467-026-70395-2)
Supplement: Supplementary file 1 — Supplementary Information [file 41467_2026_70395_MOESM1_ESM.pdf]

Mechanical Gating of Tendon Fibrogenic Transcription in Systemic Sclerosis

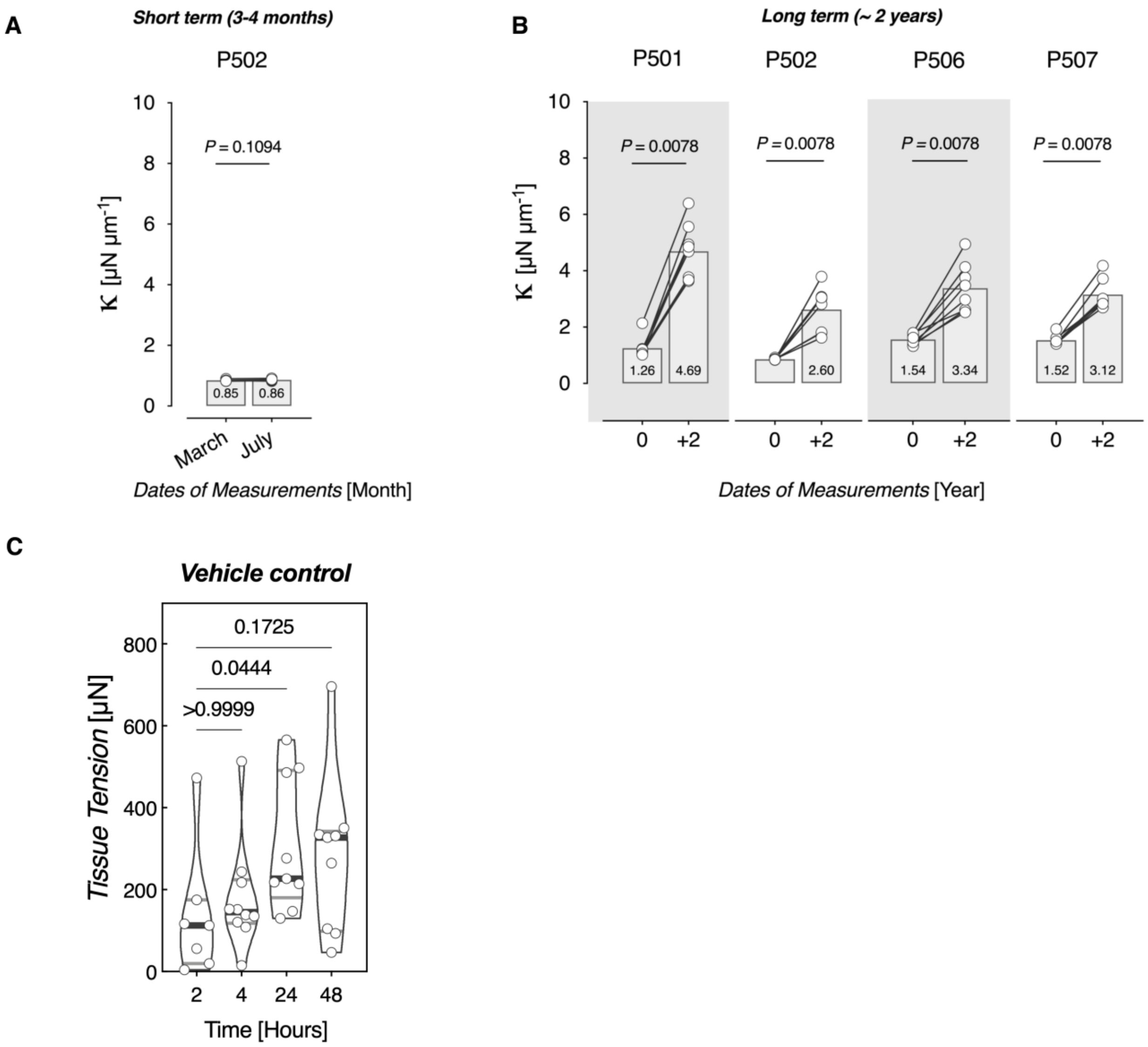

Supplementary Fig. 1 | Evaluation of the short- and long-term evolution of the steel posts spring constant (*k*)

(A-B) A well-known shortcoming of Sylgard 184 PDMS is that it contains high amounts of silica impurities which interfere with heat curing, especially when mixed in non-stoichiometric ratios (*e.g.* 50:1 or 100:1). We addressed this limitation by re-calibrating the posts spring constant at a 3 to 4-month interval. (n = 8 posts/plate). Nonparametric, Two-tailed Wilcoxon matched-pairs signed rank test. **(C)** Quantification of tissue tension of rat tail-derived tendon stromal cells in Vehicle control condition. $n \geq 7$ tissues/time-point. Unpaired, Kruskal-Wallis test with Dunn's *post-hoc* test. This data is also shown in Figure 1G.

4

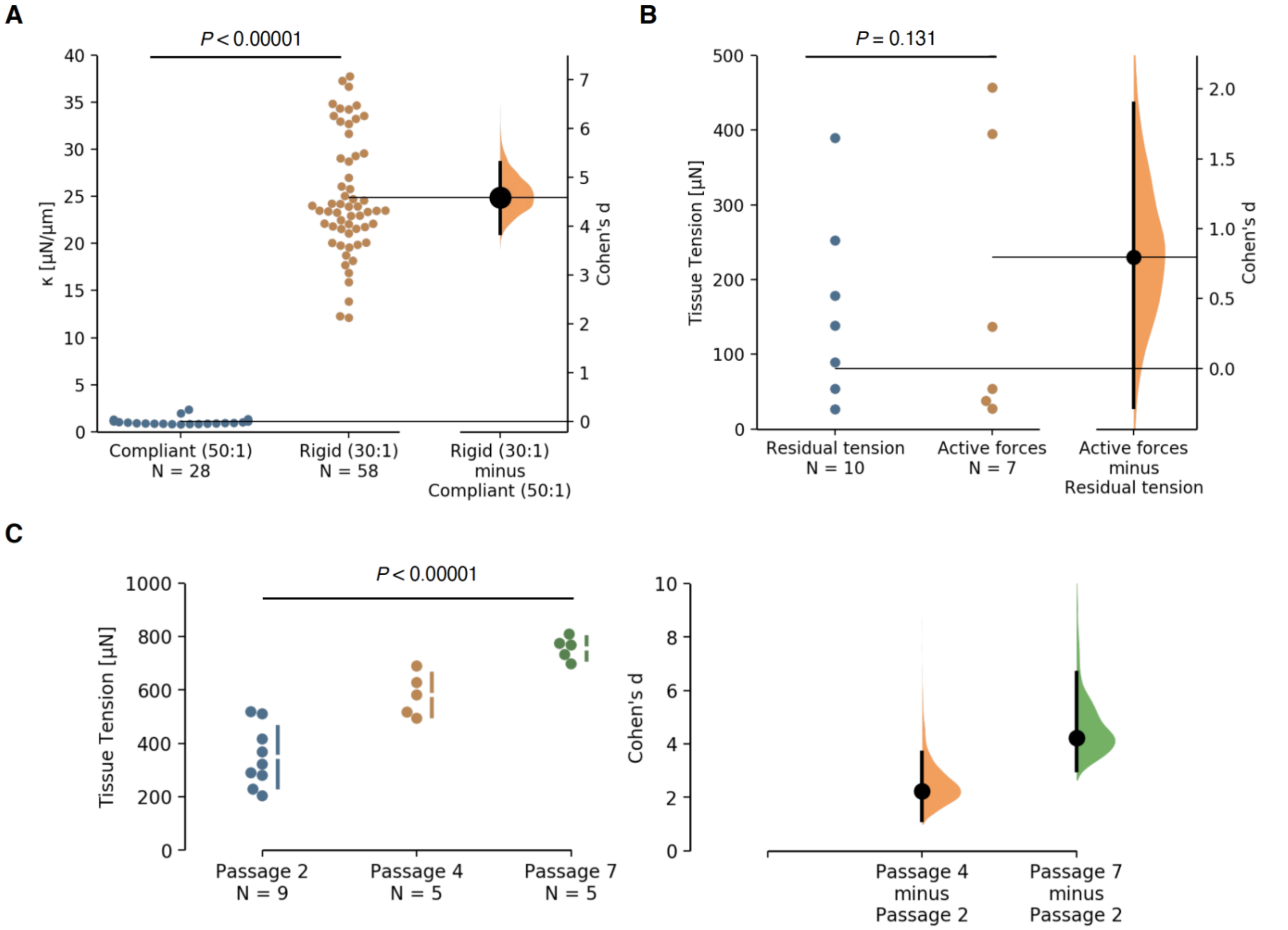

Supplementary Fig. 2 | Gardner–Altman plots for the estimation statistics for data in Figure 1.
The difference axis of the estimation plot displays the effect size, here the Cohen's *d*. The effect sizes and CIs are reported above as: effect size [CI width lower bound; upper bound]. The 95% confidence interval of Cohen's *d* is illustrated by the black vertical line. The curve displays the distribution of 5000 bootstrap re-samplings. *P* value denotes the two-sided permutation. **(A)** Post spring constant (*k*). The unpaired Cohen's d between Compliant (50:1) and Rigid (30:1) is 4.59 [95.0%CI 3.85, 5.3]. **(B)** Active and residual tension at the end of the 48h timepoint of vehicle vs stimulants conditions (n = 7-10 tissues). The unpaired Cohen's d between Residual tension and Active forces is 0.797 [95.0%CI -0.278, 1.9]. **(C)** Quantification of tissue tension as a function of the passage number of rat tail-derived tendon stromal cells (n = 5-9 tissues). The unpaired Cohen's d between Passage 2 and Passage 4 is 2.24 [95.0%CI 1.13, 3.67]. The unpaired Cohen's d between Passage 2 and Passage 7 is 4.23 [95.0%CI 3.0, 6.68].

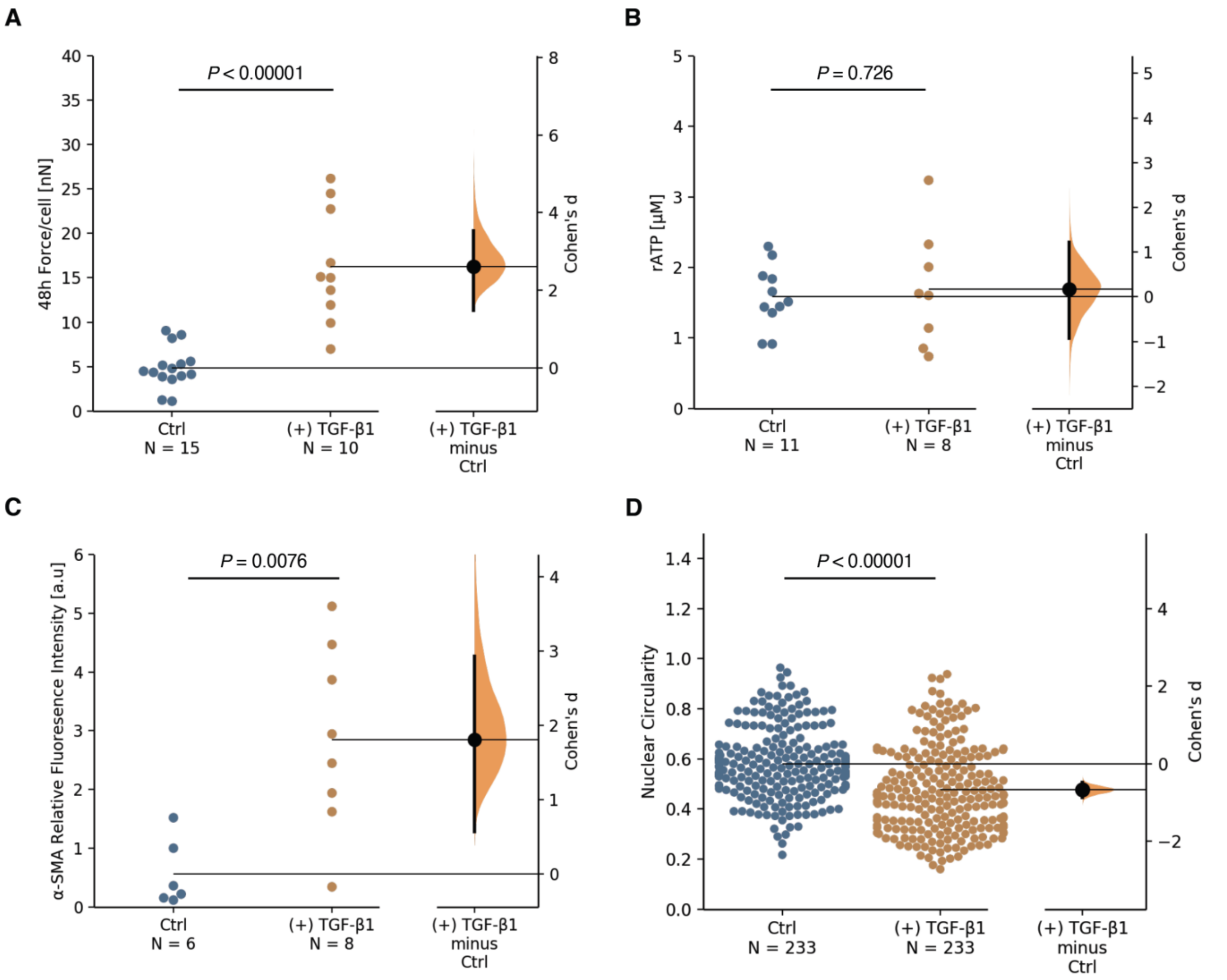

Supplementary Fig. 3 | Gardner–Altman plots for the estimation statistics for data in Figure 2.
The difference axis of the estimation plot displays the effect size, here the Cohen's *d*. The effect sizes and CIs are reported above as: effect size [CI width lower bound; upper bound]. The 95% confidence interval of Cohen's *d* is illustrated by the black vertical line. The curve displays the distribution of 5000 bootstrap re-samplings. *P* value denotes the two-sided permutation. **(A)** Quantitative analysis of tissue traction forces, as force per cell. (*n* = 10-15 tissues). The unpaired Cohen's d between Ctrl and (+) TGF-β1 is 2.61 [95.0%CI 1.48, 3.52]. **(B)** Analysis of cellular metabolic activity, as a measure of viability. (*n* = 8-11 tissues). The unpaired Cohen's d between Ctrl and (+) TGF-β1 is 0.168 [95.0%CI -0.924, 1.21]. **(C)** Quantification of fluorescent intensity levels of smooth muscle alpha-actin (α-SMA) in TGF-β1 stimulated and vehicle control. (*n* = 6-8 tissues from 2 biologically independent experiments). The unpaired Cohen's d between Ctrl and (+) TGF-β1 is 1.8 [95.0%CI 0.57, 2.93]. **(D)** Quantification of nuclear circularity of tendon-derived stromal cells. The unpaired Cohen's d between Ctrl and (+) TGF-β1 is -0.674 [95.0%CI -0.87, -0.48].

6

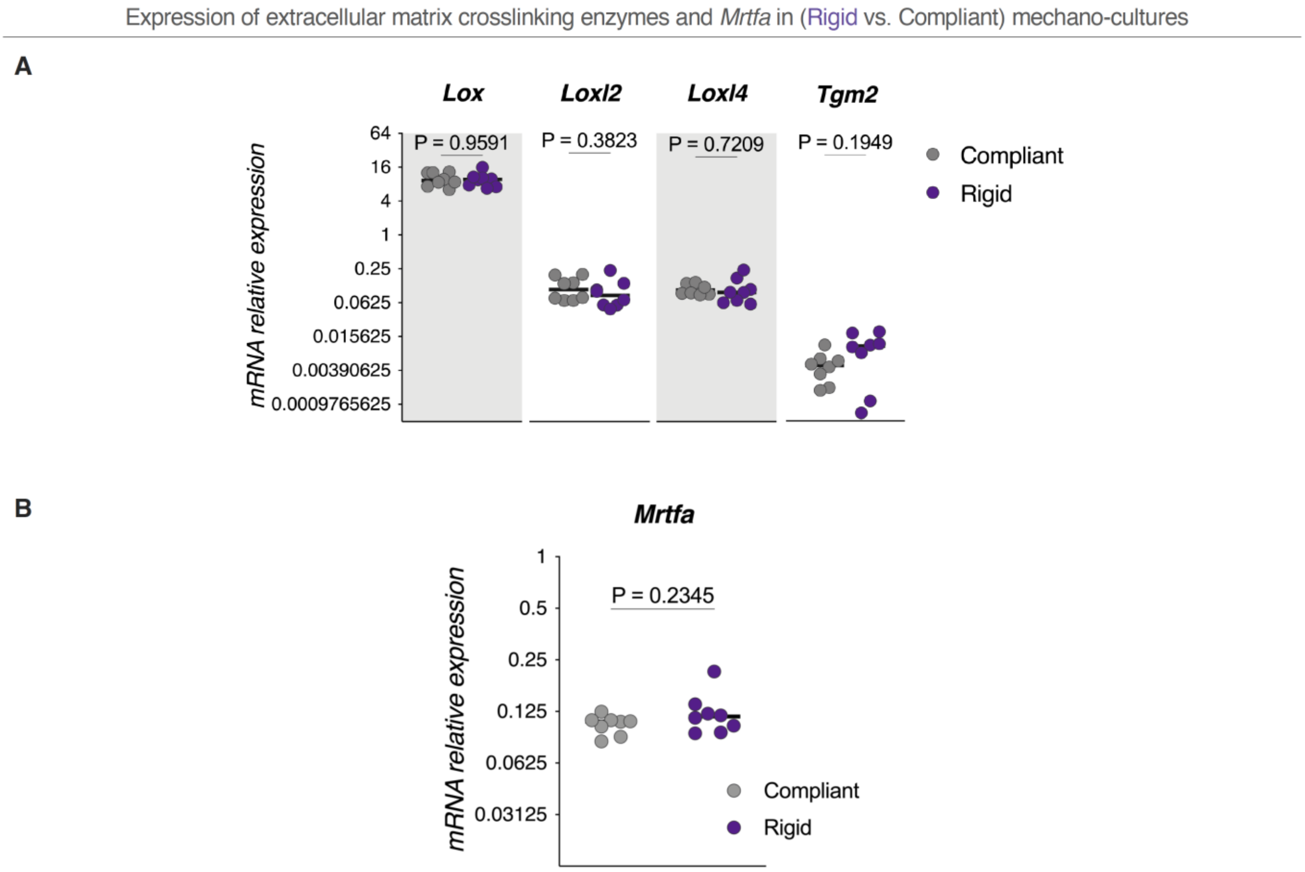

Supplementary Fig. 4 | Expression of matrix crosslinking enzymes in tensioned hydrogels.
mRNA expression of **(A)** ECM crosslinking enzymes, and **(B)** *Mrtfa* gene in rat tail tendon stromal cells tethered to different mechanical rigidities. (n = 8 replicates/group from 3 independent experiments, with each data point representing a ΔCt value of 2-3 pooled tissues). Horizontal lines indicate the median. Two-tailed, Mann Whitney test. All individual gene expression is shown normalized to *Eif4a2* and *Gapdh* reference genes. Estimation plots and permuted *P* values are in (Supplementary Fig. 6). Cohen's *d* effect sizes and CIs are reported above as: Cohen's *d* [CI width lower bound; upper bound].

8

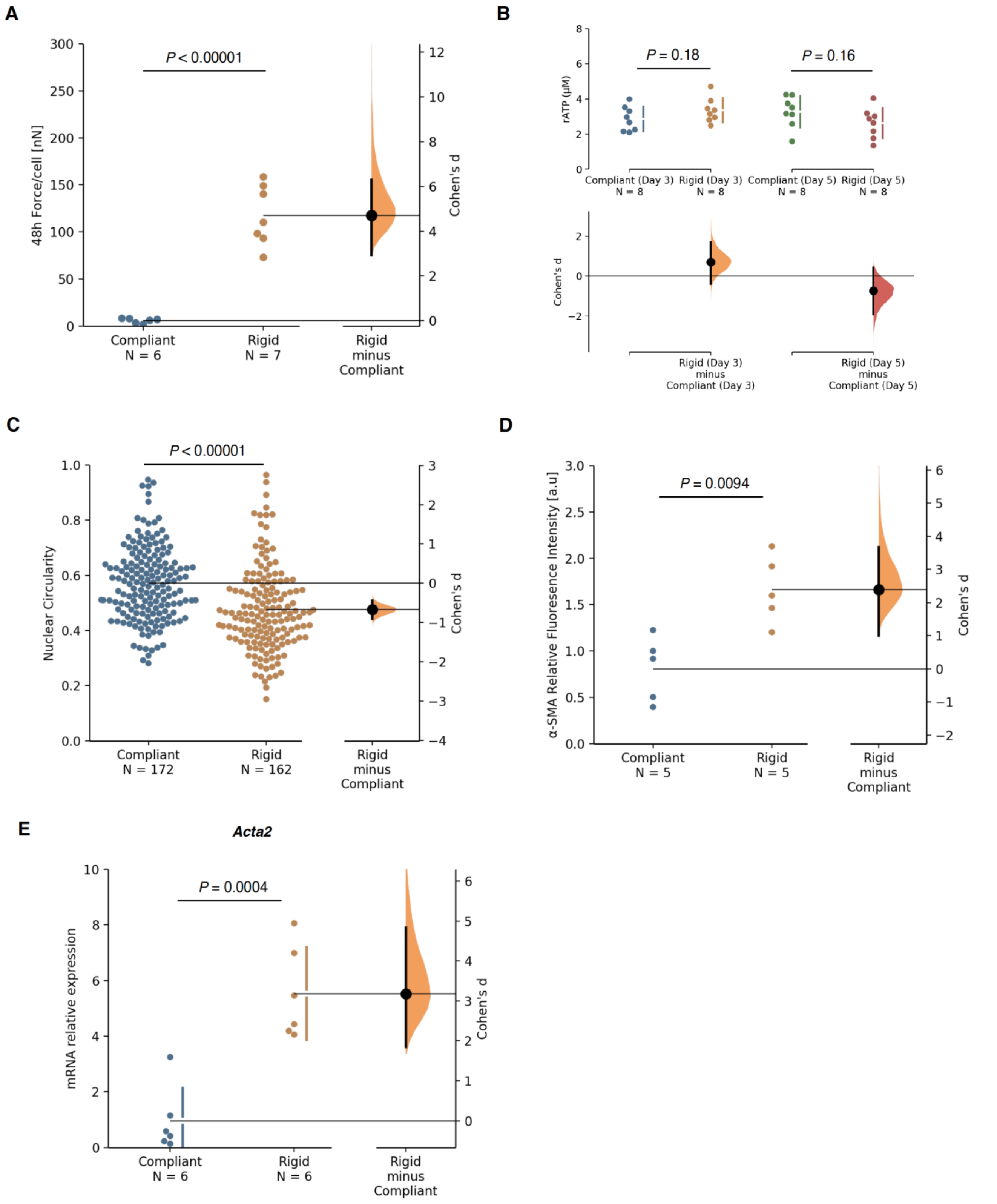

Supplementary Fig. 5 | Gardner–Altman plots for the estimation statistics for data in Figure 3

The difference axis of the estimation plot displays the effect size, here the Cohen's *d*. The effect sizes and CIs are reported above as: effect size [CI width lower bound; upper bound]. The 95% confidence interval of Cohen's *d* is illustrated by the black vertical line. The curve displays the distribution of 5000 bootstrap re-samplings. *P* value denotes the two-sided permutation. **(A)** Quantitative analysis of tissue traction forces, as force per cell (n = 6-7 tissues). The unpaired Cohen's d between Compliant and Rigid is 4.7 [95.0%CI 2.93, 6.31]. **(B)** Analysis of cellular metabolic activity, as a measure of

viability. (*n* = 8 tissues/group from 2 biologically independent experiments). The unpaired Cohen's d between Compliant_ Day3 and Rigid_ Day3 is 0.692 [95.0%CI -0.396, 1.7]. The unpaired Cohen's d between Compliant_ Day5 and Rigid_ Day5 is -0.74 [95.0%CI -1.91, 0.413]. **(C)** Quantification of nuclear circularity of tendon-derived stromal cells. The unpaired Cohen's d between Compliant and Rigid is -0.671 [95.0%CI -0.903, -0.442]. **(D)** Quantification of fluorescent intensity levels of smooth muscle alpha-actin (α-SMA), as a function of boundary stiffness. (*n* = 5 tissues from 2 biologically independent experiments). The unpaired Cohen's d between Compliant and Rigid is 2.39 [95.0%CI 0.999, 3.67]. **(E)** mRNA expression of *Acta2* gene in stromal cells tethered to different mechanical rigidities. (*n* = 6 replicates/group from 3 biologically independent experiments, with each data point representing a ΔCt value of 2-3 pooled tissues. The unpaired Cohen's d between *Acta2* Compliant and Rigid is 3.18 [95.0%CI 1.85, 4.84].

A

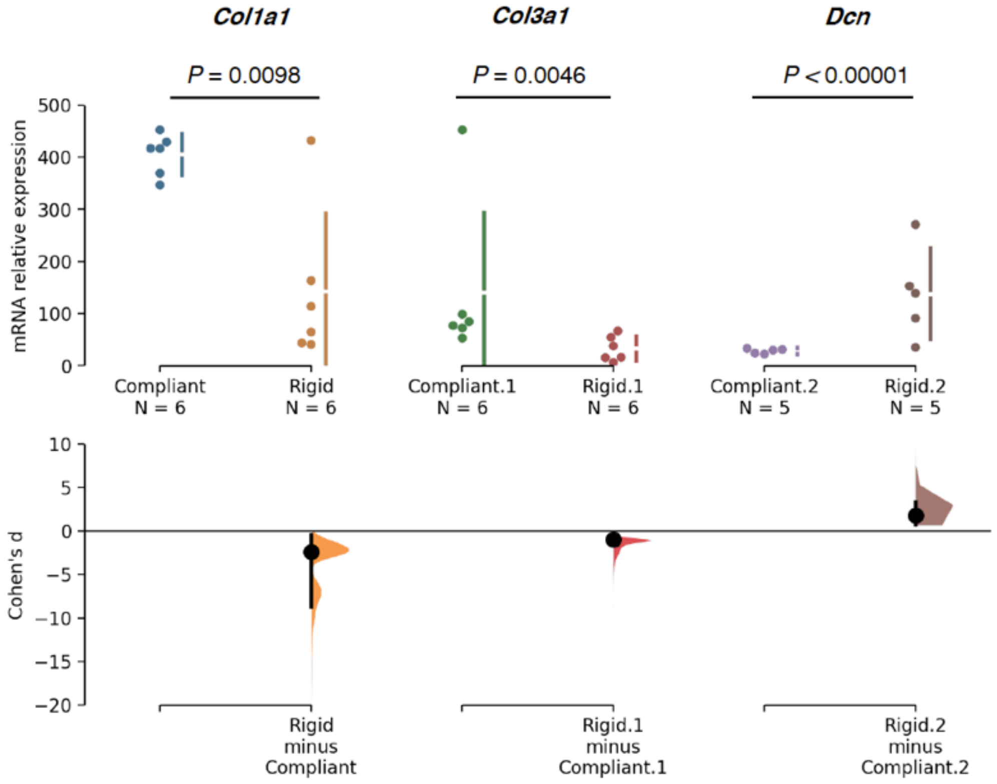

Col1a1
Col3a1
Dcn
P = 0.0098
P = 0.0046
P < 0.00001
mRNA relative expression
500
400
300
200
100
0
Compliant
N = 6
Rigid
N = 6
Compliant.1
N = 6
Rigid.1
N = 6
Compliant.2
N = 5
Rigid.2
N = 5
Cohen's d
10
5
0
−5
−10
−15
−20
Rigid
minus
Compliant
Rigid.1
minus
Compliant.1
Rigid.2
minus
Compliant.2

B

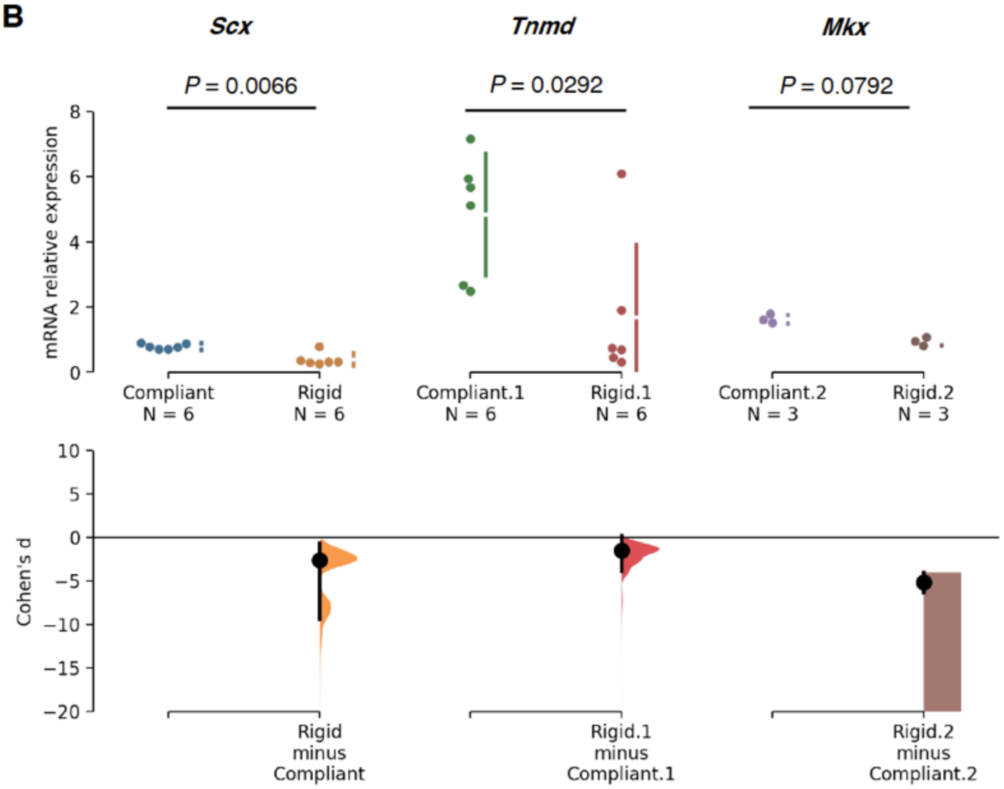

Scx
Tnmd
Mkx
P = 0.0066
P = 0.0292
P = 0.0792
mRNA relative expression
8
6
4
2
0
Compliant
N = 6
Rigid
N = 6
Compliant.1
N = 6
Rigid.1
N = 6
Compliant.2
N = 3
Rigid.2
N = 3
Cohen's d
10
5
0
−5
−10
−15
−20
Rigid
minus
Compliant
Rigid.1
minus
Compliant.1
Rigid.2
minus
Compliant.2

Supplementary Fig. 6 | Gardner–Altman plots for the estimation statistics for data in Figure 3 (continued)

The difference axis of the estimation plot displays the effect size, here the Cohen's *d*. The effect sizes and CIs are reported above as: effect size [CI width lower bound; upper bound]. The 95% confidence interval of Cohen's *d* is illustrated by the black vertical line. The curve displays the distribution of 5000 bootstrap re-samplings. *P* value denotes the two-sided permutation. **(A)** mRNA expression of ECM-related genes, and **(B)** tendon lineage-related genes in stromal cells tethered to different mechanical rigidities. (*n* = 3-6 replicates/group from 3 biologically independent experiments, with each data point representing a ΔCt value of 2-3 pooled tissues. The unpaired Cohen's d between Col1a1 Compliant and Rigid is -2.4 [95.0%CI -8.76, -0.442]. The unpaired Cohen's d between Col3a1 Compliant.1 and Rigid.1 is -0.969 [95.0%CI -1.28, -0.597]. The unpaired Cohen's d between Dcn Compliant.2 and Rigid.2 is 1.77 [95.0%CI 0.716, 3.32]. The unpaired Cohen's d between Scx Compliant and Rigid is -2.66 [95.0%CI -9.4, -0.706]. The unpaired Cohen's d between Tnmd Compliant.1 and Rigid.1 is -1.53 [95.0%CI -3.87, 0.204]. The unpaired Cohen's d between Mkx Compliant.2 and Rigid.2 is -5.14 [95.0%CI -6.32, -4.02].

A

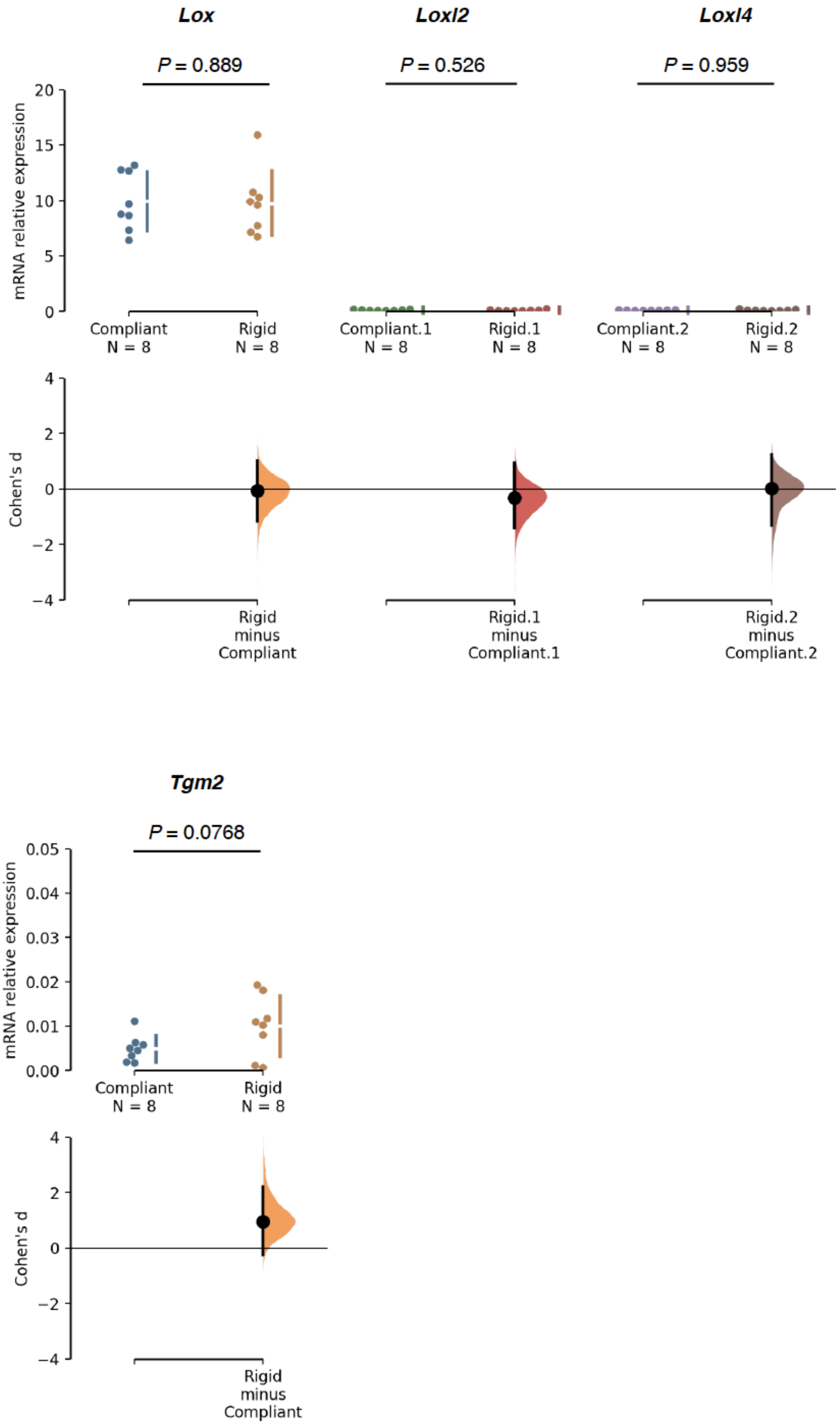

Lox
Loxl2
Loxl4
P = 0.889
P = 0.526
P = 0.959
mRNA relative expression
20
15
10
5
0
Compliant
N = 8
Rigid
N = 8
Compliant.1
N = 8
Rigid.1
N = 8
Compliant.2
N = 8
Rigid.2
N = 8
Cohen's d
4
2
0
−2
−4
Rigid
minus
Compliant
Rigid.1
minus
Compliant.1
Rigid.2
minus
Compliant.2
Tgm2
P = 0.0768
mRNA relative expression
0.05
0.04
0.03
0.02
0.01
0.00
Compliant
N = 8
Rigid
N = 8
Cohen's d
4
2
0
−2
−4
Rigid
minus
Compliant

Supplementary Fig. 6 | Gardner–Altman plots for the estimation statistics for data in Figure 3 (continued)

The difference axis of the estimation plot displays the effect size, here the Cohen's *d*. The effect sizes and CIs are reported above as: effect size [CI width   lower bound; upper bound]. The 95% confidence interval of Cohen's *d* is illustrated by the black vertical line. The curve displays the distribution of 5000 bootstrap re-samplings. *P* value denotes the two-sided permutation. **(A)** mRNA expression of ECM crosslinking genes in stromal cells tethered to different mechanical rigidities. (*n* = 8 replicates/group from 3 biologically independent experiments, with each data point representing a ΔCt value of 2-3 pooled tissues. The unpaired Cohen's *d* between *Lox* Compliant and Rigid is -0.0664 [95.0%CI -1.15, 1.01]. The unpaired Cohen's d between *Loxl2* Compliant.1 and Rigid.1 is -0.321 [95.0%CI -1.4, 0.926]. The unpaired Cohen's d between *Loxl4* Compliant.2 and Rigid.2 is 0.0271 [95.0%CI -1.31, 1.23]. The unpaired Cohen's d between *Tgm2* Compliant and Rigid is 0.958 [95.0%CI -0.249, 2.2].

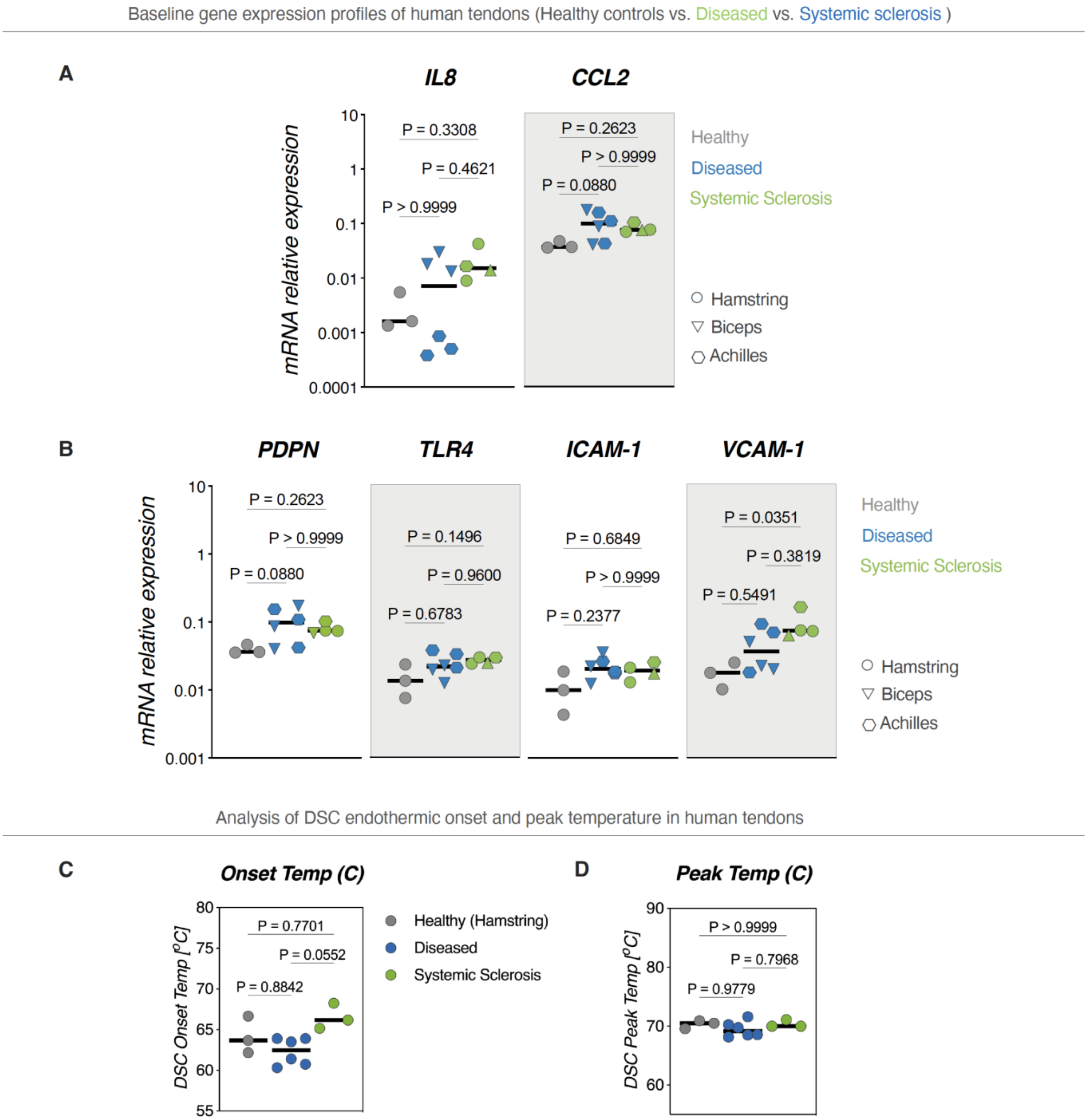

Supplementary Fig. 7 | Baseline characterization of gene expression and DSC endothermic profiles of human tendons. (A-B) Baseline mRNA expression of fibro-inflammatory activation genes in healthy (hamstring), diseased tendinopathic (biceps and Achilles) tendons, and different tendons from the SSc donor. Data points represent independent donors (n = 3 donors (healthy), 6 donors (diseased), except for the SSc condition where each datapoint is a different tendon sourced from the SSc donor (n = 4 different anatomical sites). Kruskal-Wallis test with Dunn's multiple comparisons *post-hoc* test. Horizontal lines indicate median values. All individual gene expression is shown normalized to *RPL13A* and *GAPDH* reference genes. **(C)** Endothermic onset temperature (°C), **(D)** Peak temperature of thermally-denatured human tendons as measured by DSC. Unpaired, Non-parametric Kruskal-Wallis test. Data points represent independent donors (n = 3 donors (healthy), 6 donors (diseased), except for the SSc condition where each datapoint is a different tendon sourced from the SSc donor (n = 3 different anatomical sites). Kruskal-Wallis test with Dunn's multiple comparisons *post-hoc* test. Horizontal lines indicate median values.

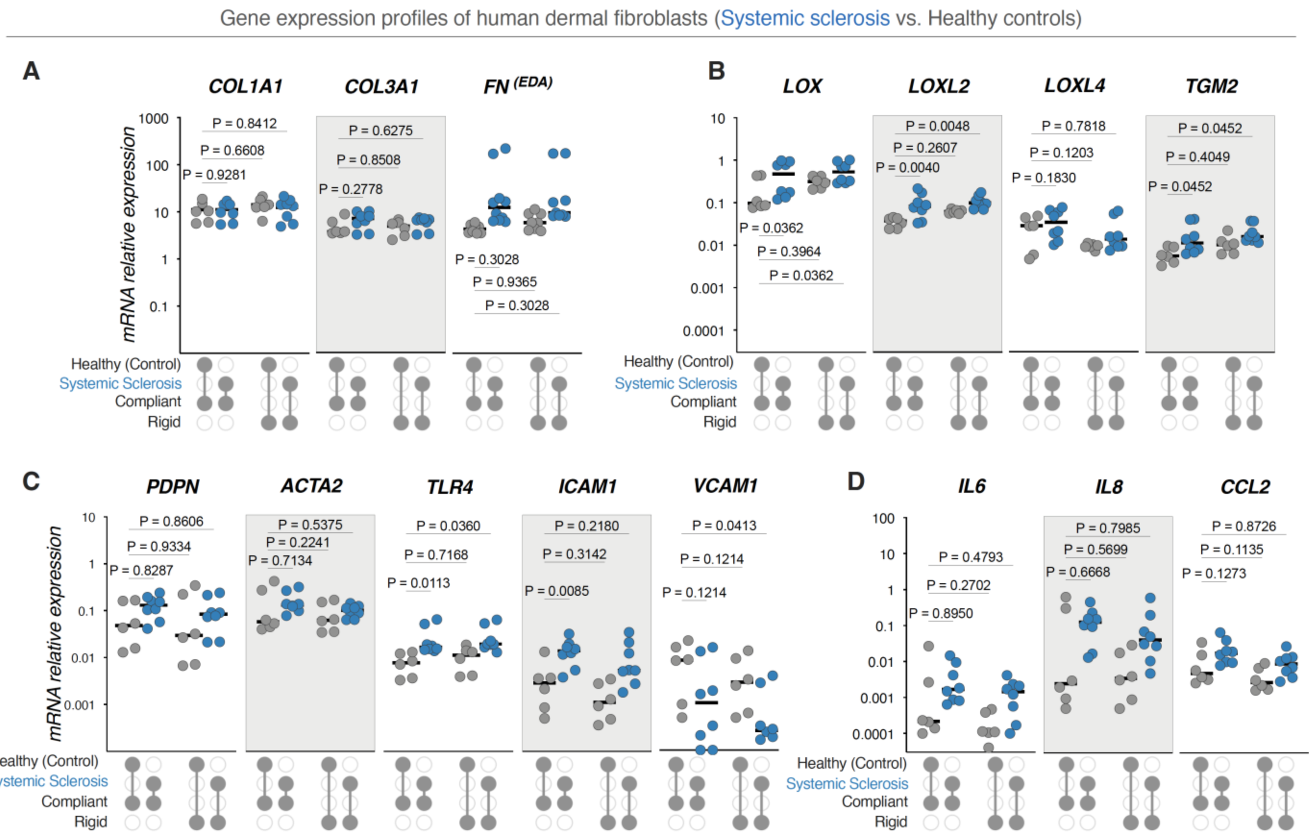

Supplementary Fig. 8 | Mechano-culture of systemic sclerosis-derived dermal stromal fibroblasts. (A) Quantitative analysis of tissue traction forces of tendon (top) and dermal (bottom) fibroblasts. Left insets: Evolution of tissue traction forces as function of time. Right insets: Violin plots of forces per cell, following normalization to initial seeding density. Horizontal lines indicate the median and interquartile range. **(B)** mRNA expression of ECM-related genes, **(C)** cross-links related enzymes **(D)** stromal activation markers, and **(E)** immune/inflammatory genes in dermal fibroblasts tethered to different mechanical rigidities. (*n* = 6 tissues (Healthy), *n* = 8 tissues (Systemic sclerosis) from 3 independent experiments, with each data point representing ΔCt. Horizontal lines indicate the median. Two-way ANOVA (boundary stiffness, disease stage) with Holm-Sidak *post-hoc* test. All individual gene expression is shown normalized to *RPL13A* and *GAPDH* reference genes.

14

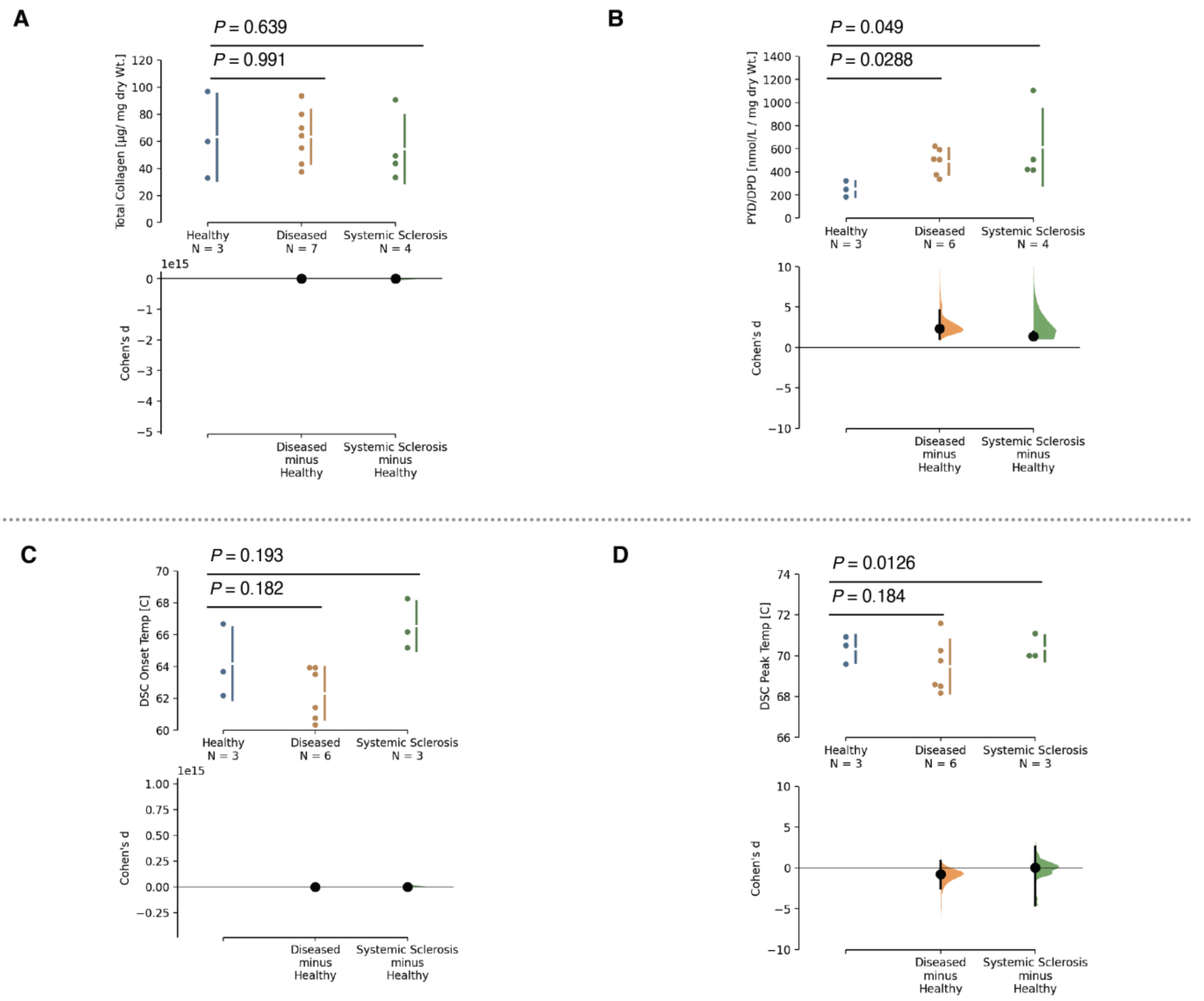

Supplementary Fig. 9 | Gardner–Altman plots for the estimation statistics for data in Figure 4.
The difference axis of the estimation plot displays the effect size, here the Cohen's d. The effect sizes and CIs are reported above as: effect size [CI width lower bound; upper bound]. The 95% confidence interval of Cohen's d is illustrated by the black vertical line. The curve displays the distribution of 5000 bootstrap re-samplings. P value denotes the two-sided permutation. **(A)** Quantification of hydroxyproline content in snap frozen tendons normalized by tissue dry weight in healthy (hamstring), diseased tendinopathic (biceps and Achilles) tendons, and different tendons from the SSc donor. The unpaired Cohen's *d* between Healthy and Diseased is 0.0064 [95.0%CI -2.25, 1.86]. The unpaired Cohen's d between Healthy and Systemic Sclerosis is -0.319 [95.0%CI -2.91, 1.63]. **(B)** Total amounts of mature trivalent crosslinks (PYD and DPD) normalized by tissue dry weight. The unpaired Cohen's *d* between Healthy and Diseased is 2.31 [95.0%CI 1.08, 4.55]. The unpaired Cohen's d between Healthy and Systemic Sclerosis is 1.39 [95.0%CI 0.997, 1.94]. **(C)** Endothermic onset temperature (°C), and **(D)** Peak temperature of thermally-denatured human tendons as measured by DSC. Onset Temp: unpaired Cohen's d between Healthy and Diseased is -1.0 [95.0%CI -2.94, 0.734]. The unpaired Cohen's d between Healthy and Systemic Sclerosis is 1.2 [95.0%CI -2.86, 4.3]. Peak Temp: The unpaired Cohen's d between Healthy and Diseased is -0.739 [95.0%CI -2.48, 0.838]. The unpaired Cohen's d between Healthy and Systemic Sclerosis is 0.0424 [95.0%CI -4.57, 2.58].

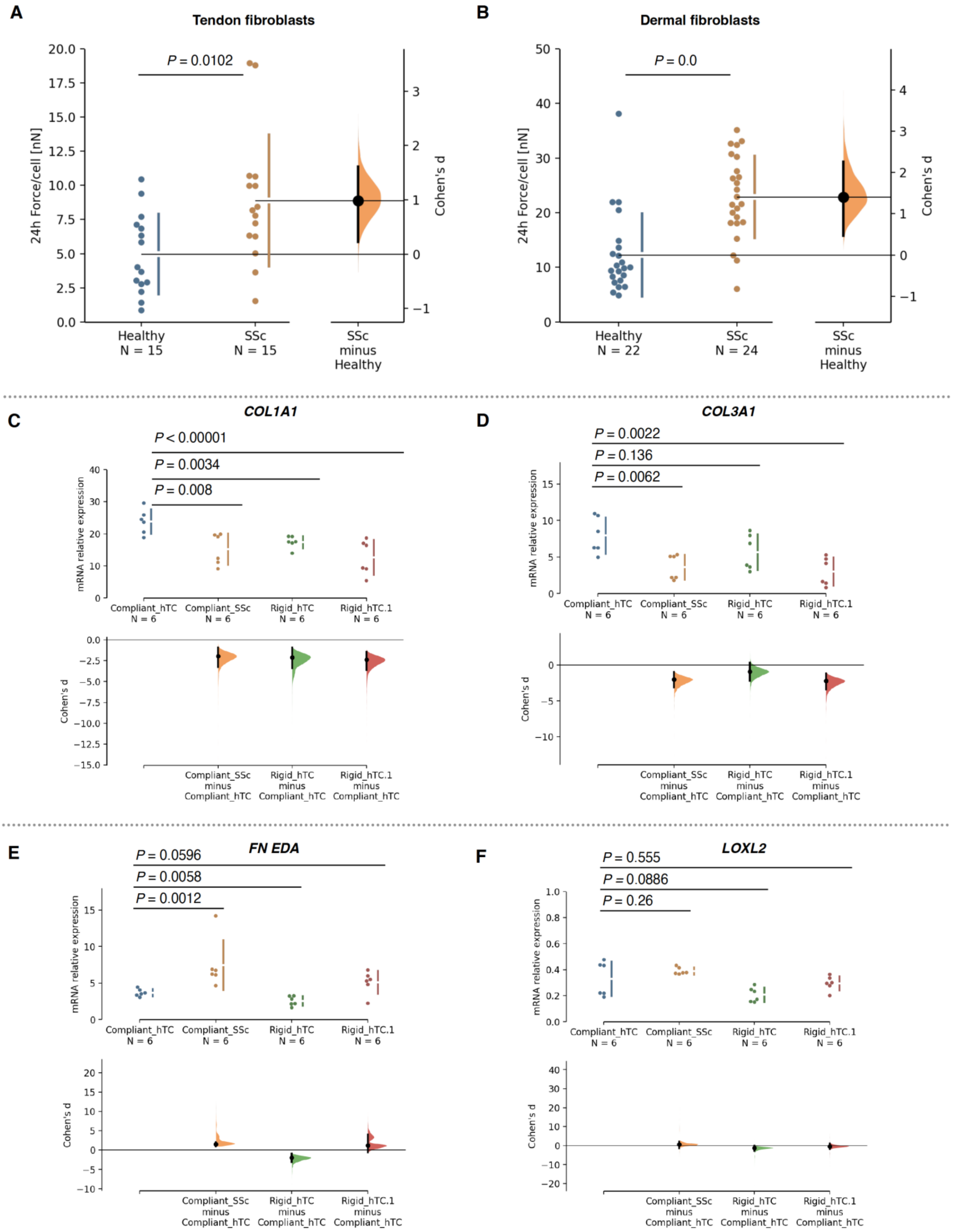
A
Tendon fibroblasts
P = 0.0102
24h Force/cell [nN]
Cohen's d
Healthy
N = 15
SSc
N = 15
SSc
minus
Healthy
B
Dermal fibroblasts
P = 0.0
24h Force/cell [nN]
Cohen's d
Healthy
N = 22
SSc
N = 24
SSc
minus
Healthy
C
COL1A1
P < 0.00001
P = 0.0034
P = 0.008
mRNA relative expression
Compliant_hTC
N = 6
Compliant_SSc
N = 6
Rigid_hTC
N = 6
Rigid_hTC.1
N = 6
Cohen's d
Compliant_SSc
minus
Compliant_hTC
Rigid_hTC
minus
Compliant_hTC
Rigid_hTC.1
minus
Compliant_hTC
D
COL3A1
P = 0.0022
P = 0.136
P = 0.0062
mRNA relative expression
Cohen's d
E
FN EDA
P = 0.0596
P = 0.0058
P = 0.0012
mRNA relative expression
Cohen's d
F
LOXL2
P = 0.555
P = 0.0886
P = 0.26
mRNA relative expression
Cohen's d

Supplementary Fig. 10 | Gardner–Altman plots for the estimation statistics for data in Figure 4 (continued).

The difference axis of the estimation plot displays the effect size, here the Cohen's *d*. The effect sizes and CIs are reported above as: effect size [CI width lower bound; upper bound]. The 95% confidence interval of Cohen's *d* is illustrated by the black vertical line. The curve displays the distribution of 5000 bootstrap re-samplings. *P* value denotes the two-sided permutation. **(A)** Quantitative analysis of tissue traction forces of tendon (top) and dermal (bottom) fibroblasts, as force per cell. (*n* = Tendon: 15 tissues/group | Skin: 22-24 tissues/group). The unpaired Cohen's d between Healthy and SSc is 0.987 [95.0%CI 0.223, 1.61]. Dermal Cohen's *d* = 1.41 [95.0%CI 0.468, 2.26]. mRNA expression of ECM-related genes of systemic sclerosis (SSc) and healthy (hTC) tendon-derived stromal cells tethered to different mechanical rigidities. **(B)** ***COL1A1.*** The unpaired Cohen's d between Compliant_hTC and Compliant_SSc is -1.96 [95.0%CI -3.27, -0.915]. The unpaired Cohen's d between Compliant_hTC and Rigid_hTC is -2.11 [95.0%CI -3.42, -0.936]. The unpaired Cohen's d between Compliant_hTC and Rigid_SSc is -2.38 [95.0%CI -3.61, -1.4]. **(C)** ***COL3A1.*** The unpaired Cohen's d between Compliant_hTC and Compliant_SSc is -2.02 [95.0%CI -3.09, -0.963]. The unpaired Cohen's d between Compliant_hTC and Rigid_hTC is -0.922 [95.0%CI -2.21, 0.302]. The unpaired Cohen's d between Compliant_hTC and Rigid_hTC.1 is -2.21 [95.0%CI -3.39, -1.16]. **(C)** ***FN(EDA).*** The unpaired Cohen's d between Compliant_hTC and Compliant_SSc is 1.56 [95.0%CI 0.946, 2.06]. The unpaired Cohen's d between Compliant_hTC and Rigid_hTC is -1.97 [95.0%CI -3.13, -0.875]. The unpaired Cohen's d between Compliant_hTC and Rigid_hTC.1 is 1.23 [95.0%CI -0.587, 4.03]. **(D)** ***LOXL2.*** The unpaired Cohen's d between Compliant_hTC and Compliant_SSc is 0.637 [95.0%CI -1.47, 2.16]. The unpaired Cohen's d between Compliant_hTC and Rigid_hTC is -1.2 [95.0%CI -2.75, -0.084]. The unpaired Cohen's d between Compliant_hTC and Rigid_hTC.1 is -0.331 [95.0%CI -1.72, 1.13].

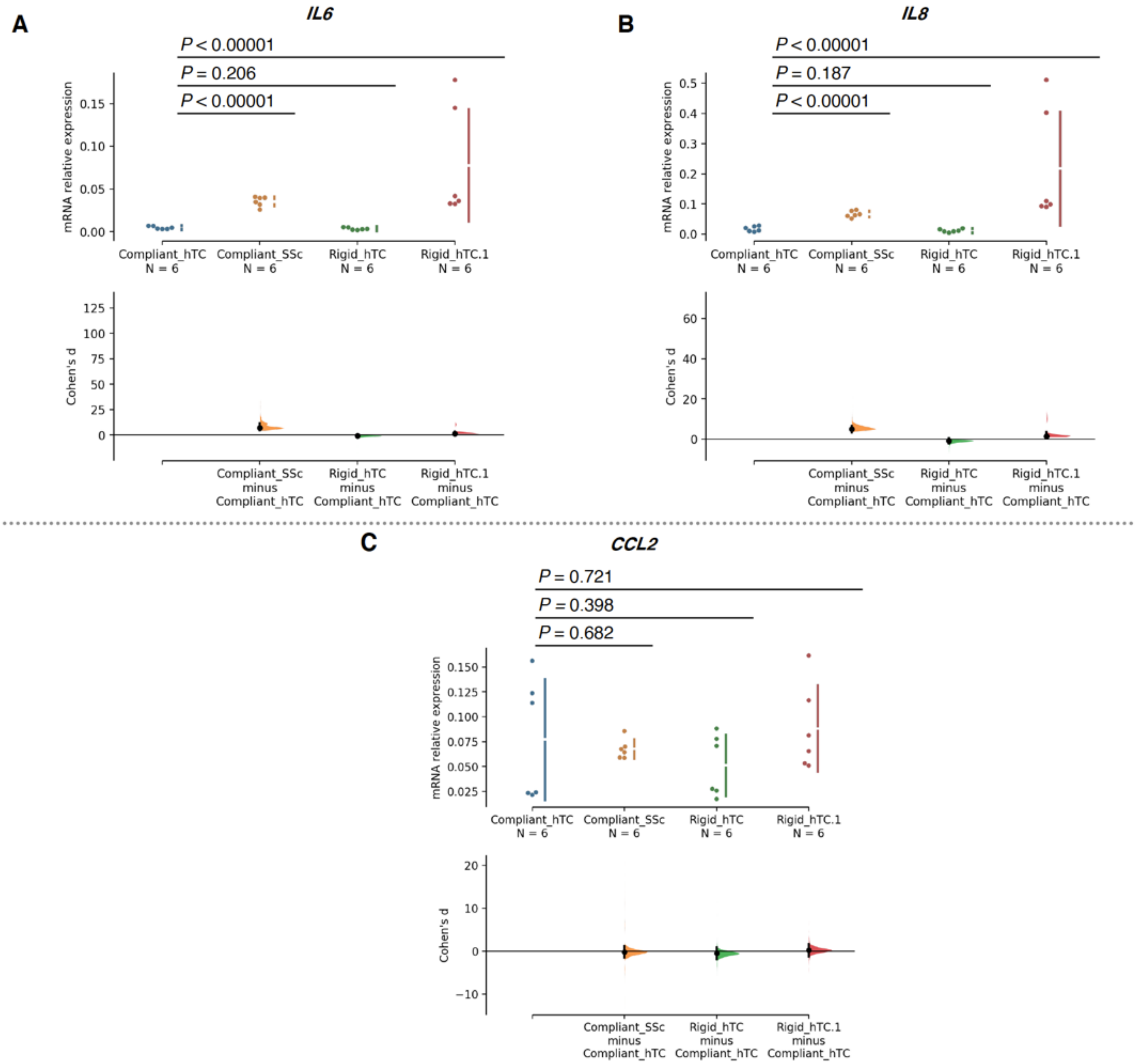

Supplementary Fig. 11 | Gardner–Altman plots for the estimation statistics for data in Figure 4 (continued)

The difference axis of the estimation plot displays the effect size, here the Cohen's *d*. The effect sizes and CIs are reported above as: effect size [CI width   lower bound; upper bound]. The 95% confidence interval of Cohen's *d* is illustrated by the black vertical line. The curve displays the distribution of 5000 bootstrap re-samplings. *P* value denotes the two-sided permutation. mRNA expression of immune/inflammatory genes of systemic sclerosis (SSc) and healthy (hTC) tendon-derived stromal cells tethered to different mechanical rigidities. **(A)** ***IL6*****.** The unpaired Cohen's d between Compliant_hTC and Compliant_SSc is 7.12 [95.0%CI 4.56, 11.9]. The unpaired Cohen's d between Compliant_hTC and Rigid_hTC is -0.784 [95.0%CI -2.1, 0.487]. The unpaired Cohen's d between Compliant_hTC and Rigid_hTC.1 is 1.57 [95.0%CI 1.25, 3.58]. **(B)** ***IL8*****.** The unpaired Cohen's d between Compliant_hTC and Compliant_SSc is 5.03 [95.0%CI 3.39, 6.55]. The unpaired Cohen's d between Compliant_hTC and Rigid_hTC is -0.824 [95.0%CI -2.14, 0.443]. The unpaired Cohen's d between Compliant_hTC and Rigid_hTC.1 is 1.5 [95.0%CI 1.17, 3.57]. **(C)** ***CCL2*****.** The unpaired Cohen's d between Compliant_hTC and Compliant_SSc is -0.22 [95.0%CI -1.55, 1.17]. The unpaired Cohen's d between Compliant_hTC and Rigid_hTC is -0.536 [95.0%CI -1.93, 0.876]. The unpaired Cohen's d between Compliant_hTC and Rigid_hTC.1 is 0.209 [95.0%CI -1.2, 1.66].

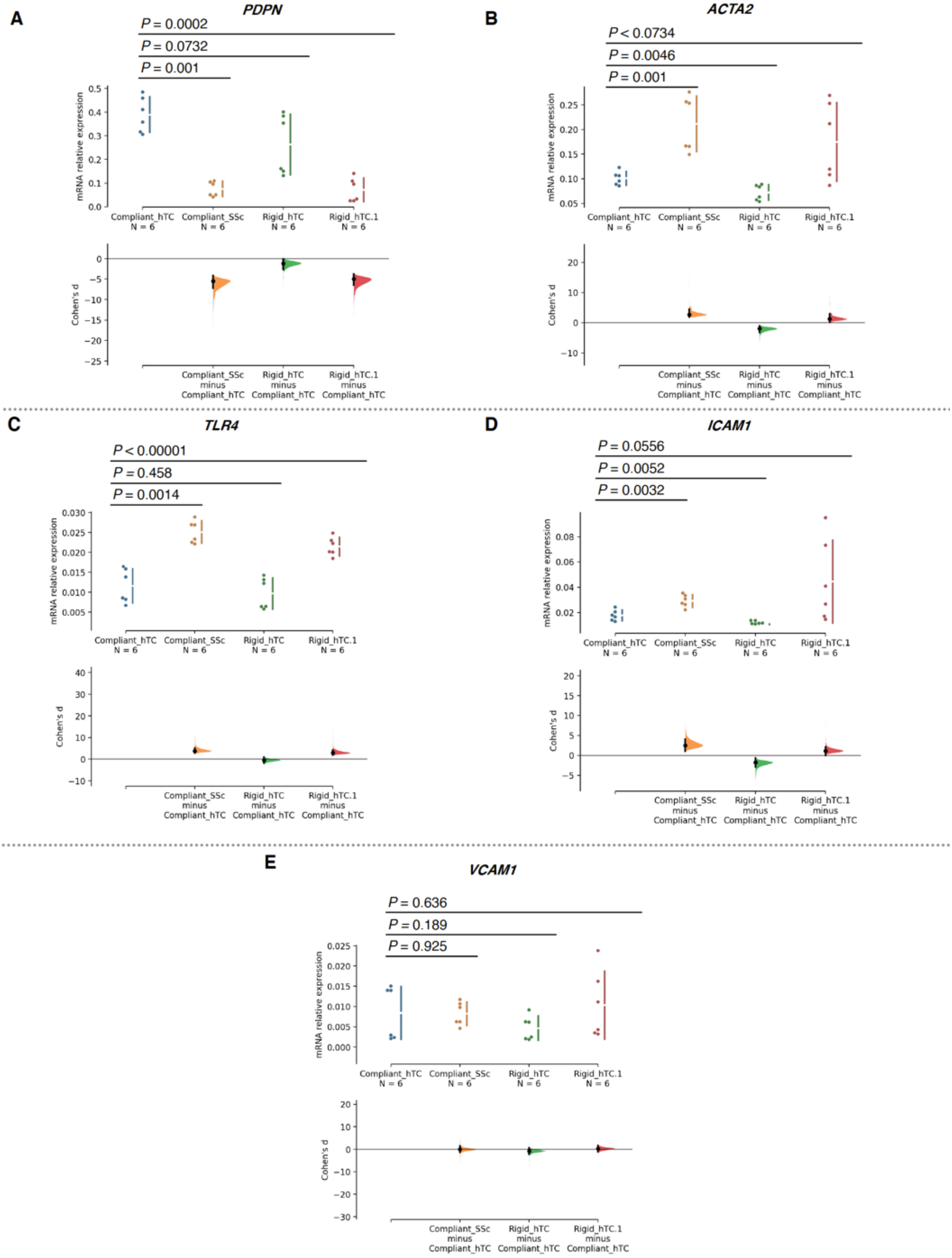
A
PDPN
P = 0.0002
P = 0.0732
P = 0.001
mRNA relative expression
Compliant_hTC N = 6
Compliant_SSc N = 6
Rigid_hTC N = 6
Rigid_hTC.1 N = 6
Cohen's d
Compliant_SSc minus Compliant_hTC
Rigid_hTC minus Compliant_hTC
Rigid_hTC.1 minus Compliant_hTC
B
ACTA2
P < 0.0734
P = 0.0046
P = 0.001
C
TLR4
P < 0.00001
P = 0.458
P = 0.0014
D
ICAM1
P = 0.0556
P = 0.0052
P = 0.0032
E
VCAM1
P = 0.636
P = 0.189
P = 0.925

Supplementary Fig. 12 | Gardner–Altman plots for the estimation statistics for data in Figure 4 (continued)

The difference axis of the estimation plot displays the effect size, here the Cohen's *d*. The effect sizes and CIs are reported above as: effect size [CI width lower bound; upper bound]. The 95% confidence interval of Cohen's *d* is illustrated by the black vertical line. The curve displays the distribution of 5000 bootstrap re-samplings. *P* value denotes the two-sided permutation. mRNA expression of stromal activation markers genes of systemic sclerosis (SSc) and healthy (hTC) tendon-derived stromal cells tethered to different mechanical rigidities. **(A)** ***PDPN.*** The unpaired Cohen's d between Compliant_hTC and Compliant_SSc is -5.49 [95.0%CI -7.2, -4.17]. The unpaired Cohen's d between Compliant_hTC and Rigid_hTC is -1.2 [95.0%CI -2.69, -0.0865]. The unpaired Cohen's d between Compliant_hTC and Rigid_hTC.1 is -5.0 [95.0%CI -6.44, -3.75]. **(B)** ***ACTA2.*** The unpaired Cohen's d between Compliant_hTC and Compliant_SSc is 2.69 [95.0%CI 1.9, 4.42]. The unpaired Cohen's d between Compliant_hTC and Rigid_hTC is -1.95 [95.0%CI -3.22, -0.998]. The unpaired Cohen's d between Compliant_hTC and Rigid_hTC.1 is 1.29 [95.0%CI 0.164, 2.88]. **(C)** ***TLR4.*** The unpaired Cohen's d between Compliant_hTC and Compliant_SSc is 3.74 [95.0%CI 2.73, 5.08]. The unpaired Cohen's d between Compliant_hTC and Rigid_hTC is -0.464 [95.0%CI -1.78, 0.805]. The unpaired Cohen's d between Compliant_hTC and Rigid_hTC.1 is 2.87 [95.0%CI 1.87, 4.21]. **(D)** ***ICAM1.*** The unpaired Cohen's d between Compliant_hTC and Compliant_SSc is 2.52 [95.0%CI 1.04, 4.09]. The unpaired Cohen's d between Compliant_hTC and Rigid_hTC is -1.77 [95.0%CI -2.88, -0.631]. The unpaired Cohen's d between Compliant_hTC and Rigid_hTC.1 is 1.15 [95.0%CI 0.0216, 2.11]. **(E)** ***VCAM1.*** The unpaired Cohen's d between Compliant_hTC and Compliant_SSc is -0.0376 [95.0%CI -1.46, 1.49]. The unpaired Cohen's d between Compliant_hTC and Rigid_hTC is -0.739 [95.0%CI -2.23, 0.617]. The unpaired Cohen's d between Compliant_hTC and Rigid_hTC.1 is 0.26 [95.0%CI -1.06, 1.63].

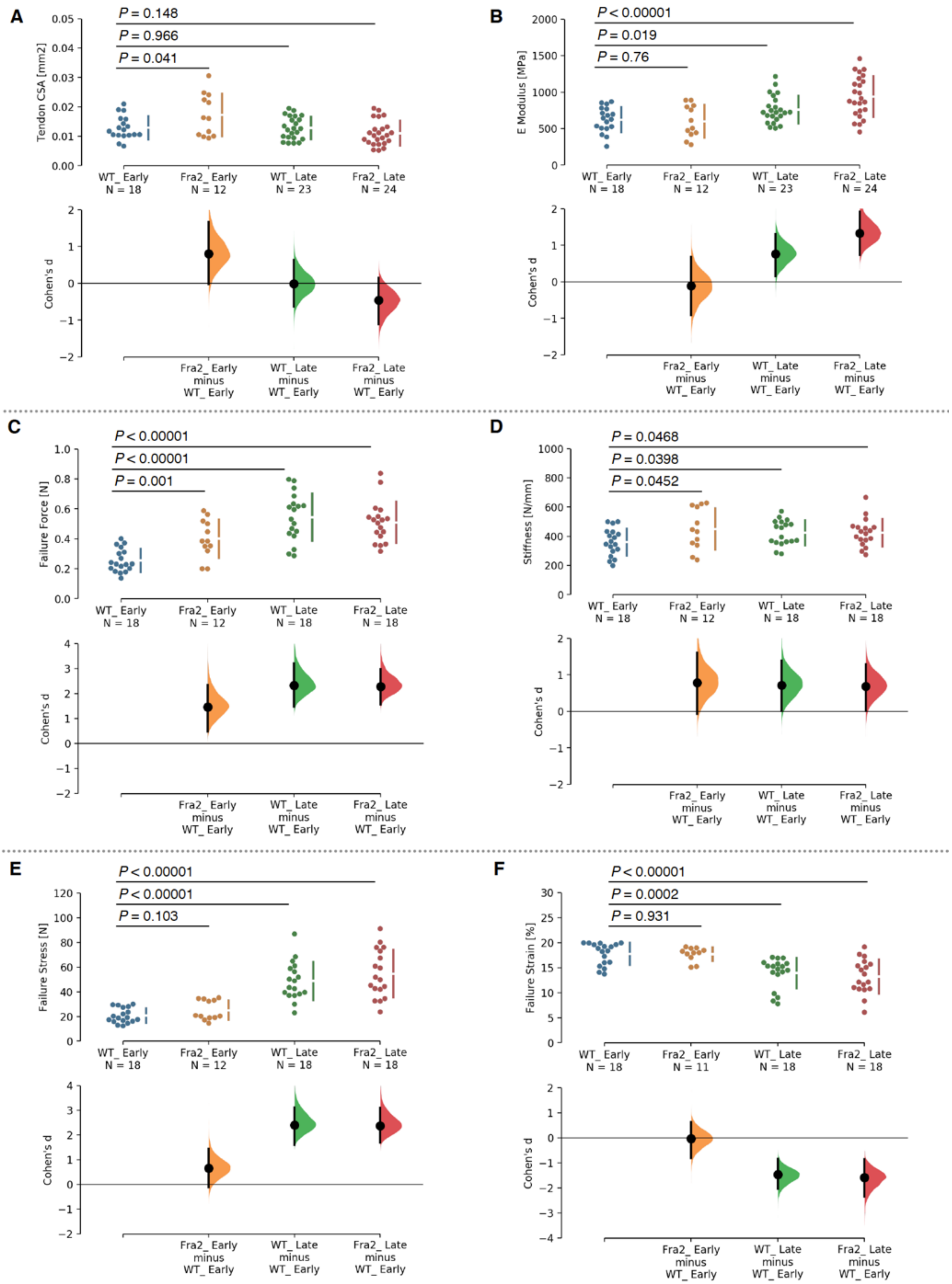
A
P = 0.148
P = 0.966
P = 0.041
Tendon CSA [mm2]
WT_ Early N = 18
Fra2_ Early N = 12
WT_ Late N = 23
Fra2_ Late N = 24
Cohen's d
Fra2_ Early minus WT_ Early
WT_ Late minus WT_ Early
Fra2_ Late minus WT_ Early
B
P < 0.00001
P = 0.019
P = 0.76
E Modulus [MPa]
WT_ Early N = 18
Fra2_ Early N = 12
WT_ Late N = 23
Fra2_ Late N = 24
Cohen's d
Fra2_ Early minus WT_ Early
WT_ Late minus WT_ Early
Fra2_ Late minus WT_ Early
C
P < 0.00001
P < 0.00001
P = 0.001
Failure Force [N]
WT_ Early N = 18
Fra2_ Early N = 12
WT_ Late N = 18
Fra2_ Late N = 18
Cohen's d
Fra2_ Early minus WT_ Early
WT_ Late minus WT_ Early
Fra2_ Late minus WT_ Early
D
P = 0.0468
P = 0.0398
P = 0.0452
Stiffness [N/mm]
WT_ Early N = 18
Fra2_ Early N = 12
WT_ Late N = 18
Fra2_ Late N = 18
Cohen's d
Fra2_ Early minus WT_ Early
WT_ Late minus WT_ Early
Fra2_ Late minus WT_ Early
E
P < 0.00001
P < 0.00001
P = 0.103
Failure Stress [N]
WT_ Early N = 18
Fra2_ Early N = 12
WT_ Late N = 18
Fra2_ Late N = 18
Cohen's d
Fra2_ Early minus WT_ Early
WT_ Late minus WT_ Early
Fra2_ Late minus WT_ Early
F
P < 0.00001
P = 0.0002
P = 0.931
Failure Strain [%]
WT_ Early N = 18
Fra2_ Early N = 11
WT_ Late N = 18
Fra2_ Late N = 18
Cohen's d
Fra2_ Early minus WT_ Early
WT_ Late minus WT_ Early
Fra2_ Late minus WT_ Early

E

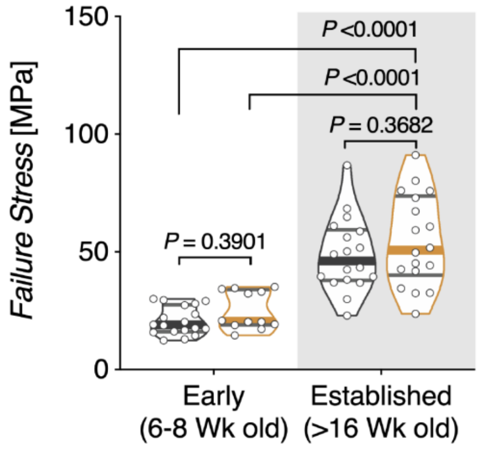

F

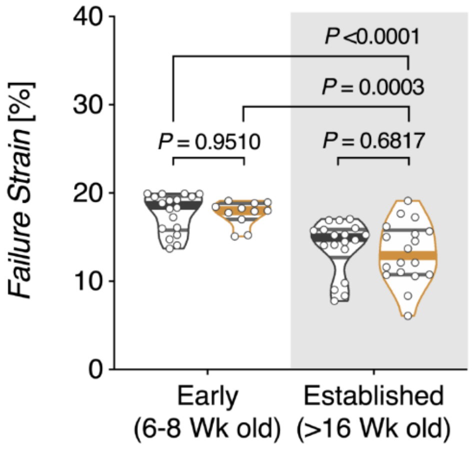

Supplementary Fig. 13 | Gardner–Altman plots for the estimation statistics for data in Figure 5.
The difference axis of the estimation plot displays the effect size, here the Cohen's *d*. The effect sizes and CIs are reported above as: effect size [CI width lower bound; upper bound]. The 95% confidence interval of Cohen's *d* is illustrated by the black vertical line. The curve displays the distribution of 5000 bootstrap re-samplings. *P* value denotes the two-sided permutation. Biomechanical characterization of Fosl-2/Fra2^{Tg} tendons and their WT littermates at early vs. late-stages of established fibrosis. Each data point represents an independent sample; (*n* = 12-24 fascicles from 6-7 mice/group). **(A) Tail tendon cross-sectional area (CSA)**. The unpaired Cohen's d between WT_ Early and Fra2_ Early is 0.8 [95.0%CI -0.0207, 1.67]. The unpaired Cohen's d between WT_ Early and WT_ Late is -0.0135 [95.0%CI -0.642, 0.633]. The unpaired Cohen's d between WT_ Early and Fra2_ Late is -0.461 [95.0%CI -1.12, 0.152]. **(B) *E* modulus**. The unpaired Cohen's d between WT_ Early and Fra2_ Early is -0.113 [95.0%CI -0.913, 0.681]. The unpaired Cohen's d between WT_ Early and WT_ Late is 0.763 [95.0%CI 0.145, 1.31]. The unpaired Cohen's d between WT_ Early and Fra2_ Late is 1.33 [95.0%CI 0.738, 1.92]. **(C) Failure force**. The unpaired Cohen's d between WT_ Early and Fra2_ Early is 1.46 [95.0%CI 0.47, 2.34]. The unpaired Cohen's d between WT_ Early and WT_ Late is 2.33 [95.0%CI 1.48, 3.21]. The unpaired Cohen's d between WT_ Early and Fra2_ Late is 2.29 [95.0%CI 1.57, 2.98]. **(D) Stiffness**. The unpaired Cohen's d between WT_ Early and Fra2_ Early is 0.784 [95.0%CI -0.0689, 1.61]. The unpaired Cohen's d between WT_ Early and WT_ Late is 0.718 [95.0%CI 0.0101, 1.39]. The unpaired Cohen's d between WT_ Early and Fra2_ Late is 0.688 [95.0%CI 0.00584, 1.3]. **(E) Failure stress**. The unpaired Cohen's d between WT_ Early and Fra2_ Early is 0.649 [95.0%CI -0.115, 1.45]. The unpaired Cohen's d between WT_ Early and WT_ Late is 2.4 [95.0%CI 1.59, 3.12]. The unpaired Cohen's d between WT_ Early and Fra2_ Late is 2.38 [95.0%CI 1.69, 3.11]. **(F) Failure strain**. The unpaired Cohen's d between WT_ Early and Fra2_ Early is -0.0332 [95.0%CI -0.8, 0.63]. The unpaired Cohen's d between WT_ Early and WT_ Late is -1.46 [95.0%CI -2.04, -0.834]. The unpaired Cohen's d between WT_ Early and Fra2_ Late is -1.58 [95.0%CI -2.34, -0.858].

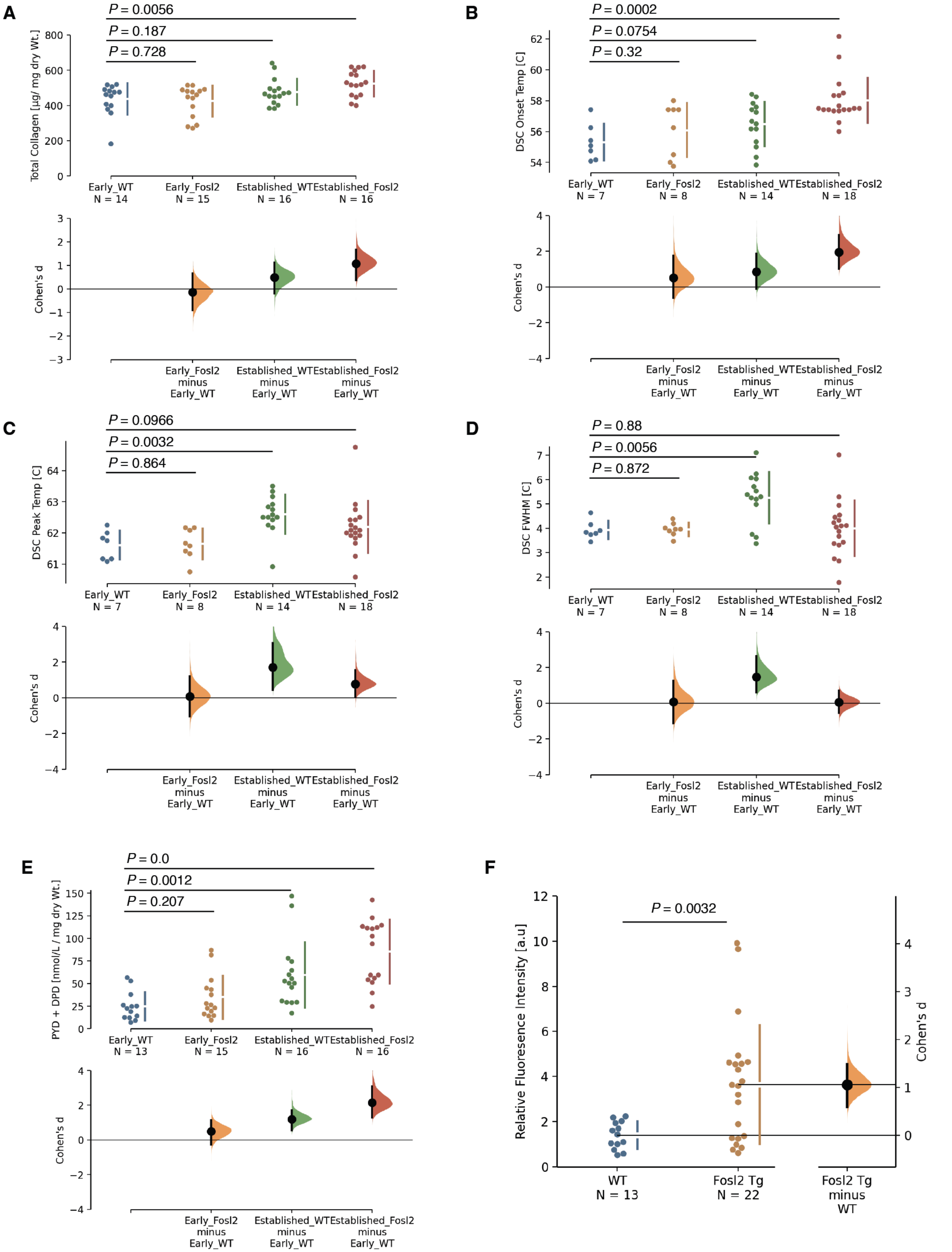
A
P = 0.0056
P = 0.187
P = 0.728
Total Collagen [µg/ mg dry Wt.]
Early_WT N = 14
Early_Fosl2 N = 15
Established_WT N = 16
Established_Fosl2 N = 16
Cohen's d
Early_Fosl2 minus Early_WT
Established_WT minus Early_WT
Established_Fosl2 minus Early_WT
B
P = 0.0002
P = 0.0754
P = 0.32
DSC Onset Temp [C]
Early_WT N = 7
Early_Fosl2 N = 8
Established_WT N = 14
Established_Fosl2 N = 18
Cohen's d
C
P = 0.0966
P = 0.0032
P = 0.864
DSC Peak Temp [C]
Early_WT N = 7
Early_Fosl2 N = 8
Established_WT N = 14
Established_Fosl2 N = 18
Cohen's d
D
P = 0.88
P = 0.0056
P = 0.872
DSC FWHM [C]
Early_WT N = 7
Early_Fosl2 N = 8
Established_WT N = 14
Established_Fosl2 N = 18
Cohen's d
E
P = 0.0
P = 0.0012
P = 0.207
PYD + DPD [nmol/L / mg dry Wt.]
Early_WT N = 13
Early_Fosl2 N = 15
Established_WT N = 16
Established_Fosl2 N = 16
Cohen's d
F
P = 0.0032
Relative Fluoresence Intensity [a.u]
Cohen's d
WT N = 13
Fosl2 Tg N = 22
Fosl2 Tg minus WT

Supplementary Fig. 14 | Gardner–Altman plots for the estimation statistics for data in Figure 5 (continued).

The difference axis of the estimation plot displays the effect size, here the Cohen's *d*. The effect sizes and CIs are reported above as: effect size [CI width lower bound; upper bound]. The 95% confidence interval of Cohen's *d* is illustrated by the black vertical line. The curve displays the distribution of 5000 bootstrap re-samplings. *P* value denotes the two-sided permutation. **(A)** Quantification of hydroxyproline content in tendons (*n* = 7 mice/genotype). The unpaired Cohen's d between WT and Fosl-2$^{(Tg)}$ is 0.771 [95.0%CI -0.353, 1.94]. Thermal denaturing of tendons as measured by DSC (*n* = 7 mice/genotype). **(B)** DSC Endothermic onset temperature (°C).The unpaired Cohen's d between WT and Fosl2 Tg is 0.889 [95.0%CI -0.376, 1.48]. **(C)** Peak temperature (°C).The unpaired Cohen's d between WT and Fosl2 Tg is -0.0761 [95.0%CI -1.22, 1.04]. **(D)** Full-width at half-maximum (FWHM). The unpaired Cohen's d between WT and Fosl2 Tg is 0.15 [95.0%CI -1.07, 1.26]. **(F)** In-situ Lox activity: The unpaired Cohen's d between WT and Fosl2 Tg is 1.06 [95.0%CI 0.588, 1.49].

22

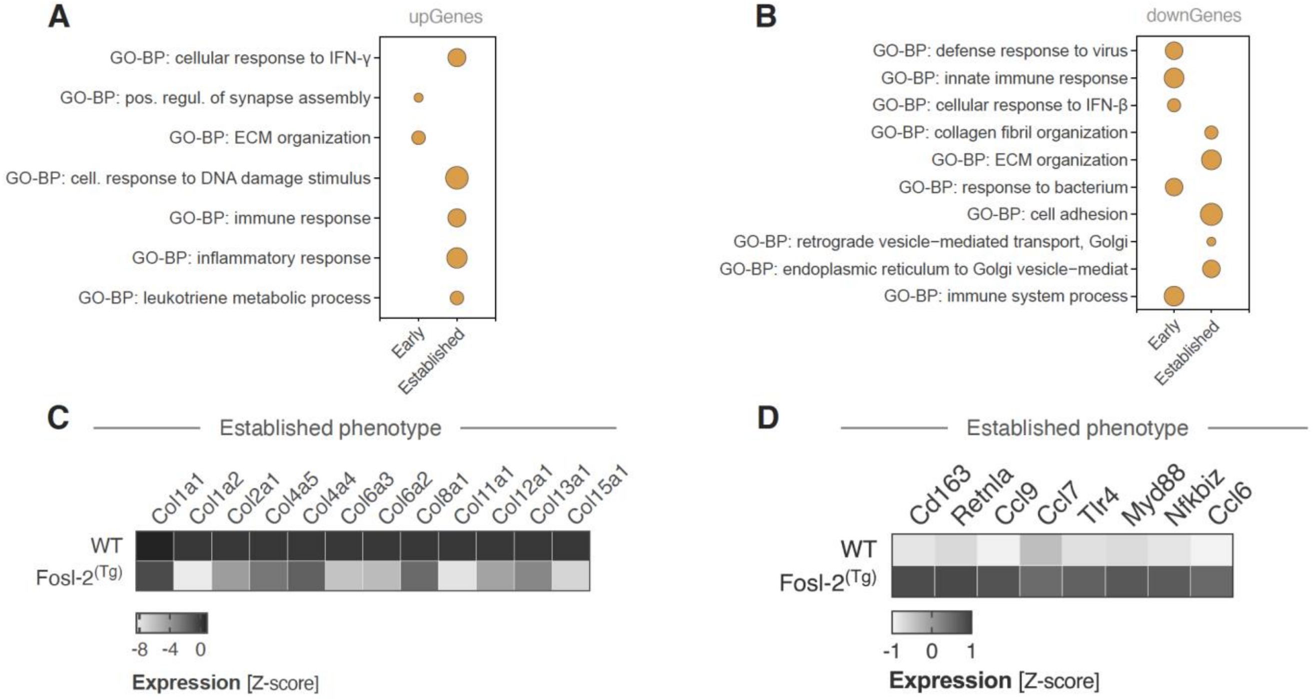

Supplementary Fig. 15 | Transcriptome of fibrotic Fosl-2$^{(Tg)}$ tendons is enriched in extracellular matrix and macrophages activation signatures.

(A-B) Pathway bubble plots depicting over-representation analysis (ORA) of multiple DEGs comparison between Fosl-2$^{(Tg)}$ *vs.* WT tendons in early and established phenotypes. (A) Upregulated genes. (B) Downregulated genes. **(C-D)** Normalized expression of collagen genes (C) and macrophage activation genes (D) in bulk RNA-sequencing of established Fosl-2$^{(Tg)}$ *vs.* wild-type tendons.

24

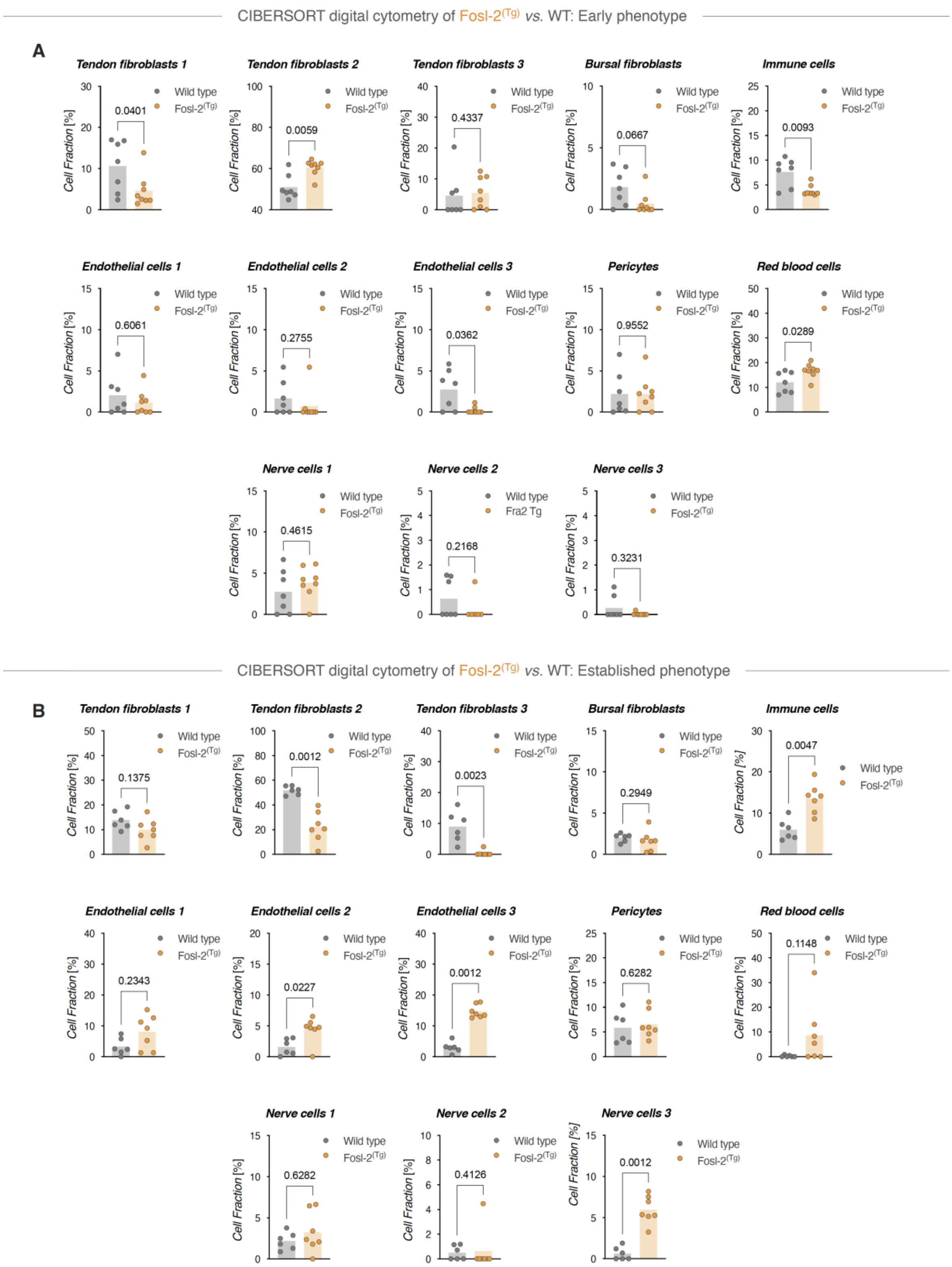

Supplementary Fig. 16 | CIBERSORT digital cytometry of Fosl-2$^{(Tg)}$ *vs.* WT tendons.

(A-B) CIBERSORT inferred composition of 13 cell fractions in the transcriptome-wide RNA-seq dataset. Dot plots showing inferred percentage of each cell type in WT and Fosl-2$^{(Tg)}$ in tendons from the Early (A) and Established (B) phenotypes. Each dot represents an independent biological unit (mouse). Statistical significance was determined using unpaired, non-parametric Mann Whitney test.

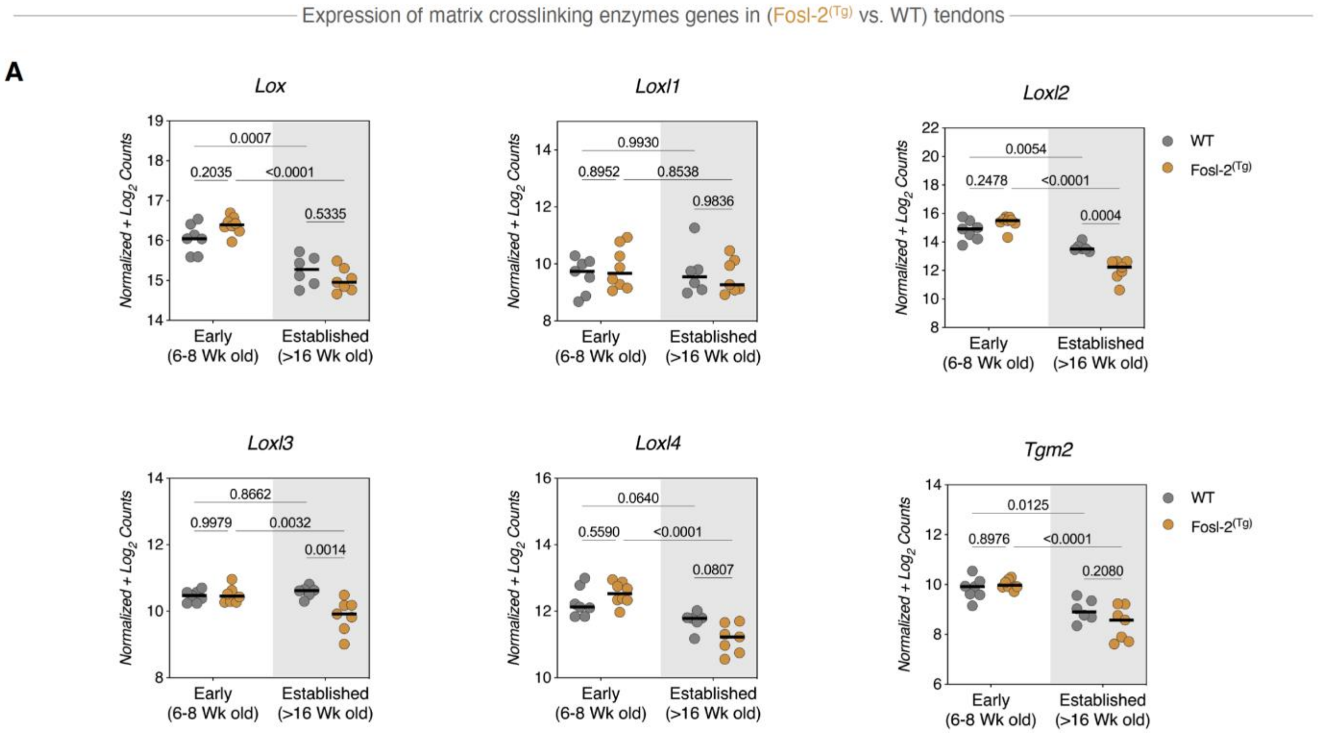

Supplementary Fig. 17 | Expression of matrix crosslinking enzymes in Fosl-2$^{(Tg)}$ mice. A) Normalized RNA-Seq expression values of matrix crosslinking enzymes in tail tendons of (Fosl-2$^{(Tg)}$ vs. Wild-type) mice. (Fosl-2$^{(Tg)}$: *n* = 7 mice – WT: *n* = 6 mice). Horizontal lines indicate the mean. ANOVA Tukey's multiple comparisons test.

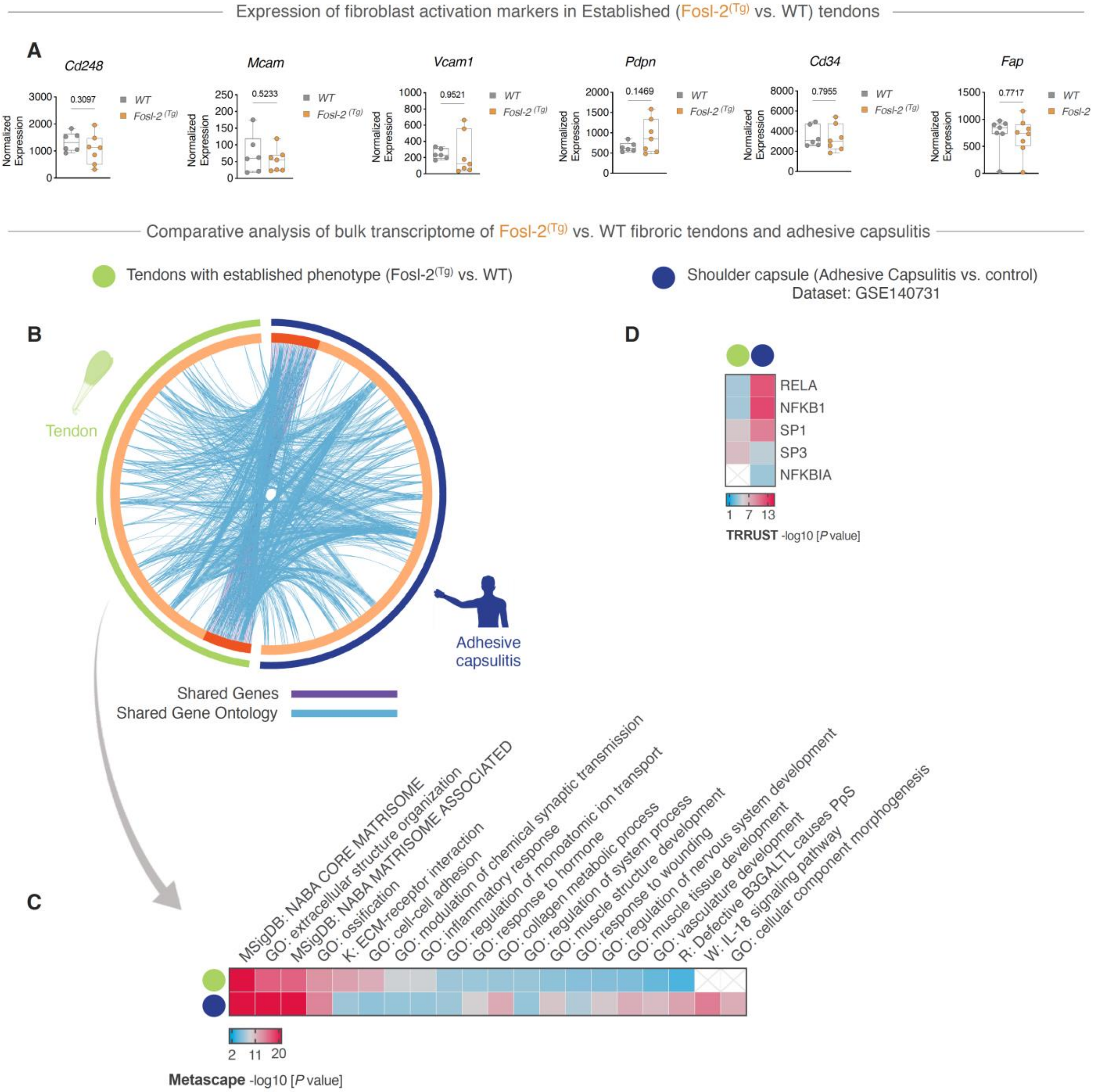

Supplementary Fig. 18 | Gene list-based comparative analysis of mouse tendons with established fibrosis with human capsular tissue with adhesive capsulitis. (A) Normalized RNA-Seq expression values of selected fibroblast activation markers in tendons with established phenotype (Fosl-2$^{(Tg)}$ vs. Wild-type). (Fosl-2$^{(Tg)}$: $n = 7$ mice – WT: $n = 6$ mice). Horizontal lines indicate the median. Welch's t-test. **(B)** Circos plot of overlapped input genes and enrichment features between the two groups. Dark orange arc and purple lines represent the differentially expressed genes that are shared between the two groups. Blue lines connect the genes that fall under the same statistically significantly enriched ontology term, reflecting the extent of functional overlap between the two comparison groups. **(C)** Heatmap of top significantly enriched terms (GO, KEGG, canonical pathways, etc.) using Metascape. Color code depicts −log10(hypergeometric p-value) of enrichment test. **(D)** Top 5 significantly enriched transcriptional regulators of shared targets in (C) using TRRUST database for transcription factor analysis.

Abbreviations: (R) Reactome, (W) WikiPathways, (K) KEGG, (H) Hallmark, (GO) Gene Ontology, (MSigDB) Molecular Signatures Database.

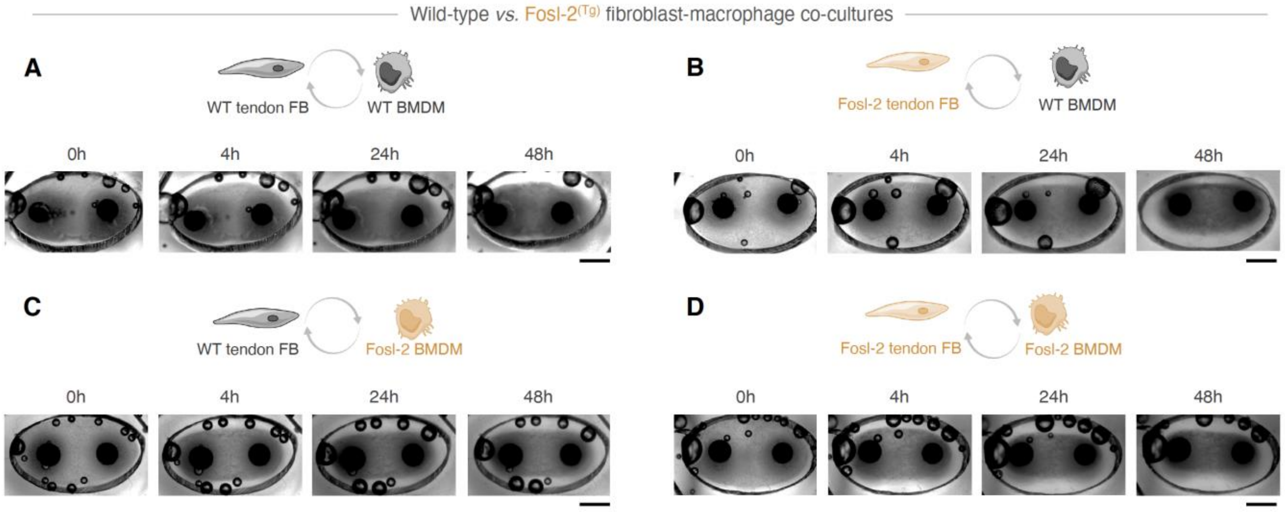

Supplementary Fig. 19 | (A-D) Representative images showing the time-course of tissue compaction in Wild-type (WT) *vs.* Fosl-2(Tg) fibroblasts-macrophages co-cultures. Scale bar = 1 mm.

26

A

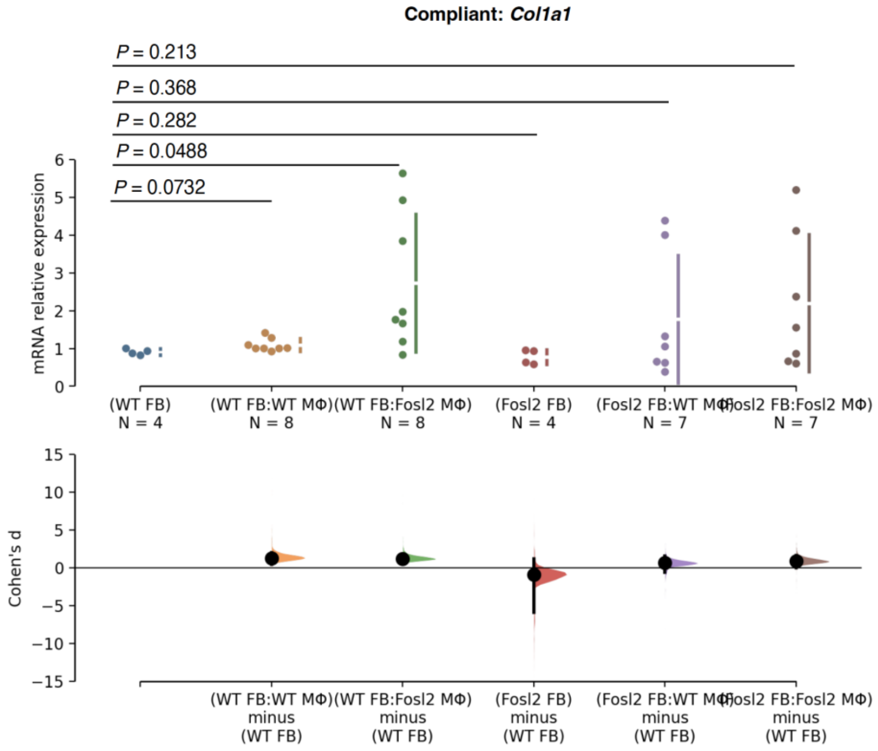

Compliant: Col1a1
P = 0.213
P = 0.368
P = 0.282
P = 0.0488
P = 0.0732
mRNA relative expression
(WT FB) N = 4
(WT FB:WT MΦ) N = 8
(WT FB:Fosl2 MΦ) N = 8
(Fosl2 FB) N = 4
(Fosl2 FB:WT MΦ) N = 7
(Fosl2 FB:Fosl2 MΦ) N = 7
Cohen's d
(WT FB:WT MΦ) minus (WT FB)
(WT FB:Fosl2 MΦ) minus (WT FB)
(Fosl2 FB) minus (WT FB)
(Fosl2 FB:WT MΦ) minus (WT FB)
(Fosl2 FB:Fosl2 MΦ) minus (WT FB)

B

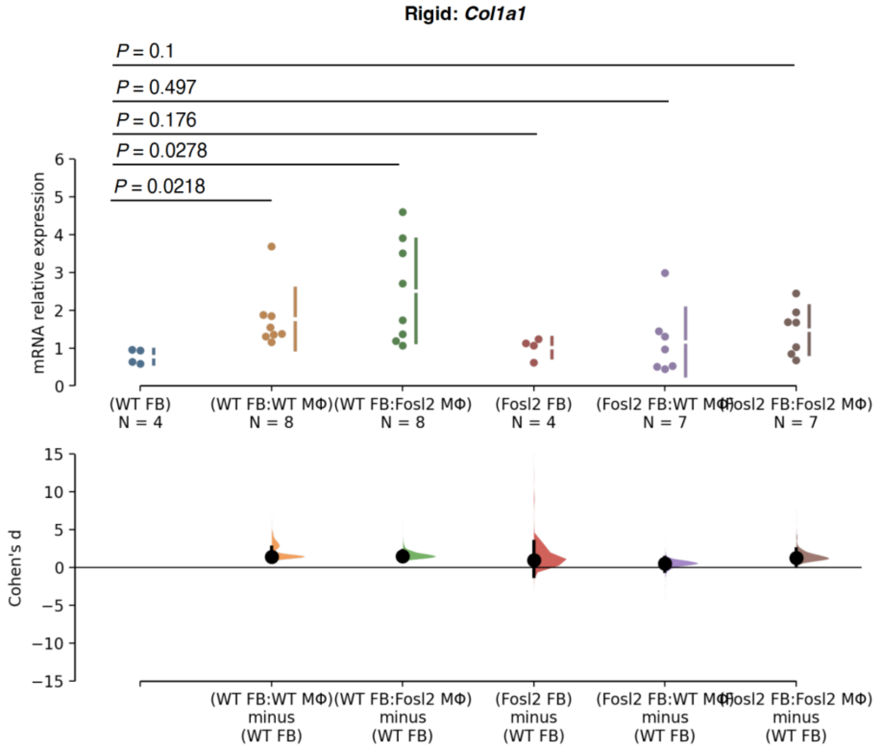

Rigid: Col1a1
P = 0.1
P = 0.497
P = 0.176
P = 0.0278
P = 0.0218
mRNA relative expression
(WT FB) N = 4
(WT FB:WT MΦ) N = 8
(WT FB:Fosl2 MΦ) N = 8
(Fosl2 FB) N = 4
(Fosl2 FB:WT MΦ) N = 7
(Fosl2 FB:Fosl2 MΦ) N = 7
Cohen's d
(WT FB:WT MΦ) minus (WT FB)
(WT FB:Fosl2 MΦ) minus (WT FB)
(Fosl2 FB) minus (WT FB)
(Fosl2 FB:WT MΦ) minus (WT FB)
(Fosl2 FB:Fosl2 MΦ) minus (WT FB)

Supplementary Fig. 20 | *Col1a1* Gardner–Altman plots for the estimation statistics for data in Figure 7D. mRNA expression of *Col1a1* gene of Fosl-2$^{(Tg)}$ and WT stromal cells in monoculture and direct cocultures tethered to different mechanical boundary rigidities. **(A)** Compliant *Col1a1*: The unpaired Cohen's d between (WT FB) and (WT FB:WT MΦ) is 1.25 [95.0%CI 0.468, 2.01]. The unpaired Cohen's d between (WT FB) and (WT FB:Fosl2 MΦ) is 1.2 [95.0%CI 0.621, 1.77]. The unpaired Cohen's d between (WT FB) and (Fosl2 FB) is -0.894 [95.0%CI -5.92, 1.17]. The unpaired Cohen's d between (WT FB) and (Fosl2 FB:WT MΦ) is 0.63 [95.0%CI -0.643, 1.55]. The unpaired Cohen's d between (WT FB) and (Fosl2 FB:Fosl2 MΦ) is 0.868 [95.0%CI 0.0, 1.64]. **(B)** Rigid *Col1a1*: The unpaired Cohen's d between (WT FB) and (WT FB:WT MΦ) is 1.43 [95.0%CI 0.813, 2.7]. The unpaired Cohen's d between (WT FB) and (WT FB:Fosl2 MΦ) is 1.51 [95.0%CI 0.855, 2.26]. The unpaired Cohen's d between (WT FB) and (Fosl2 FB) is 0.982 [95.0%CI -1.18, 3.43]. The unpaired Cohen's d between (WT FB) and (Fosl2 FB:WT MΦ) is 0.528 [95.0%CI -0.562, 1.34]. The unpaired Cohen's d between (WT FB) and (Fosl2 FB:Fosl2 MΦ) is 1.29 [95.0%CI 0.298, 2.45].

The difference axis of the estimation plot displays the effect size, here the Cohen's *d*. The effect sizes and CIs are reported above as: effect size [CI width lower bound; upper bound]. The 95% confidence interval of Cohen's *d* is illustrated by the black vertical line. The curve displays the distribution of 5000 bootstrap re-samplings. *P* value denotes the two-sided permutation.

A

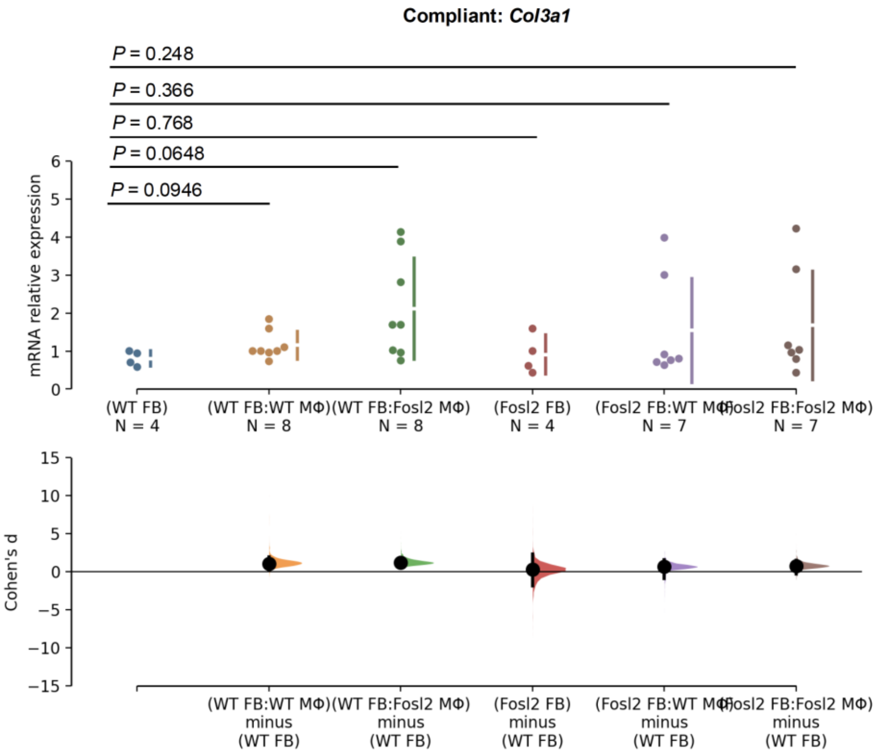
Compliant: Col3a1
P = 0.248
P = 0.366
P = 0.768
P = 0.0648
P = 0.0946
mRNA relative expression
(WT FB)
N = 4
(WT FB:WT MΦ)
N = 8
(WT FB:Fosl2 MΦ)
N = 8
(Fosl2 FB)
N = 4
(Fosl2 FB:WT MΦ)
N = 7
(Fosl2 FB:Fosl2 MΦ)
N = 7
Cohen's d
(WT FB:WT MΦ) minus (WT FB)
(WT FB:Fosl2 MΦ) minus (WT FB)
(Fosl2 FB) minus (WT FB)
(Fosl2 FB:WT MΦ) minus (WT FB)
(Fosl2 FB:Fosl2 MΦ) minus (WT FB)

B

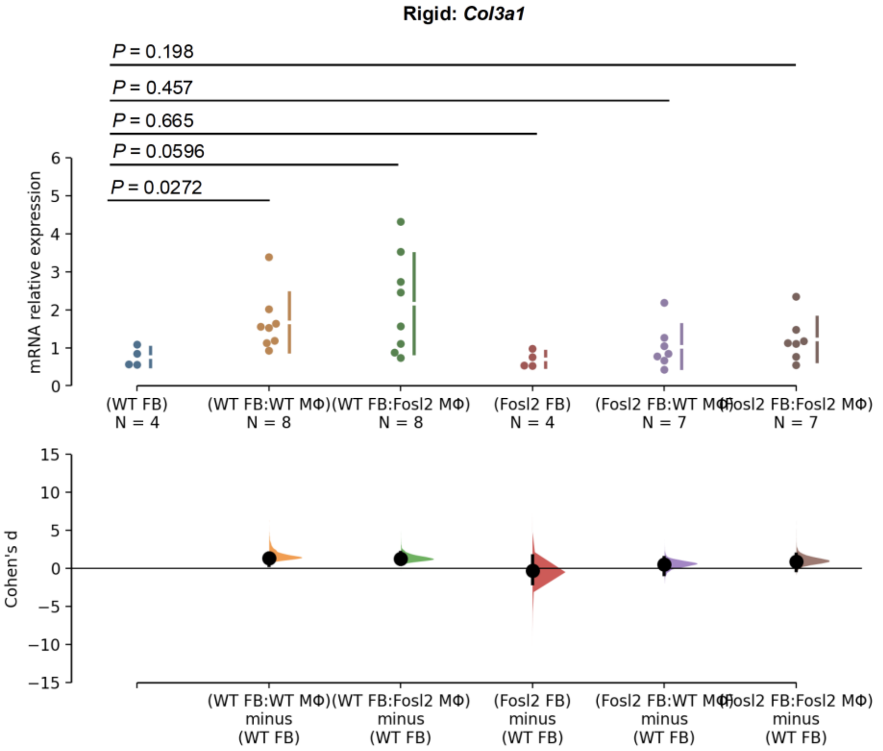
Rigid: Col3a1
P = 0.198
P = 0.457
P = 0.665
P = 0.0596
P = 0.0272
mRNA relative expression
(WT FB)
N = 4
(WT FB:WT MΦ)
N = 8
(WT FB:Fosl2 MΦ)
N = 8
(Fosl2 FB)
N = 4
(Fosl2 FB:WT MΦ)
N = 7
(Fosl2 FB:Fosl2 MΦ)
N = 7
Cohen's d
(WT FB:WT MΦ) minus (WT FB)
(WT FB:Fosl2 MΦ) minus (WT FB)
(Fosl2 FB) minus (WT FB)
(Fosl2 FB:WT MΦ) minus (WT FB)
(Fosl2 FB:Fosl2 MΦ) minus (WT FB)

Supplementary Fig. 21 | *Col3a1* Gardner–Altman plots for the estimation statistics for data in Figure 7D. mRNA expression of *Col3a1* gene of Fosl-2$^{(Tg)}$ and WT stromal cells in monoculture and direct cocultures tethered to different mechanical boundary rigidities. **(A)**Compliant *Co31a1*: The unpaired Cohen's d between (WT FB) and (WT FB:WT MΦ) is 1.06 [95.0%CI 0.148, 1.94]. The unpaired Cohen's d between (WT FB) and (WT FB:Fosl2 MΦ) is 1.17 [95.0%CI 0.523, 1.82]. The unpaired Cohen's d between (WT FB) and (Fosl2 FB) is 0.263 [95.0%CI -1.88, 2.33]. The unpaired Cohen's d between (WT FB) and (Fosl2 FB:WT MΦ) is 0.658 [95.0%CI -0.875, 1.57]. The unpaired Cohen's d between (WT FB) and (Fosl2 FB:Fosl2 MΦ) is 0.745 [95.0%CI -0.305, 1.47]. **(B)** Rigid *Co31a1*: The unpaired Cohen's d between (WT FB) and (WT FB:WT MΦ) is 1.37 [95.0%CI 0.384, 2.0]. The unpaired Cohen's d between (WT FB) and (WT FB:Fosl2 MΦ) is 1.27 [95.0%CI 0.498, 2.06]. The unpaired Cohen's d between (WT FB) and (Fosl2 FB) is -0.277 [95.0%CI -2.04, 1.61]. The unpaired Cohen's d between (WT FB) and (Fosl2 FB:WT MΦ) is 0.542 [95.0%CI -0.845, 1.38]. The unpaired Cohen's d between (WT FB) and (Fosl2 FB:Fosl2 MΦ) is 0.922 [95.0%CI -0.3, 1.82].

The difference axis of the estimation plot displays the effect size, here the Cohen's *d*. The effect sizes and CIs are reported above as: effect size [CI width lower bound; upper bound]. The 95% confidence interval of Cohen's *d* is illustrated by the black vertical line. The curve displays the distribution of 5000 bootstrap re-samplings. *P* value denotes the two-sided permutation.

32

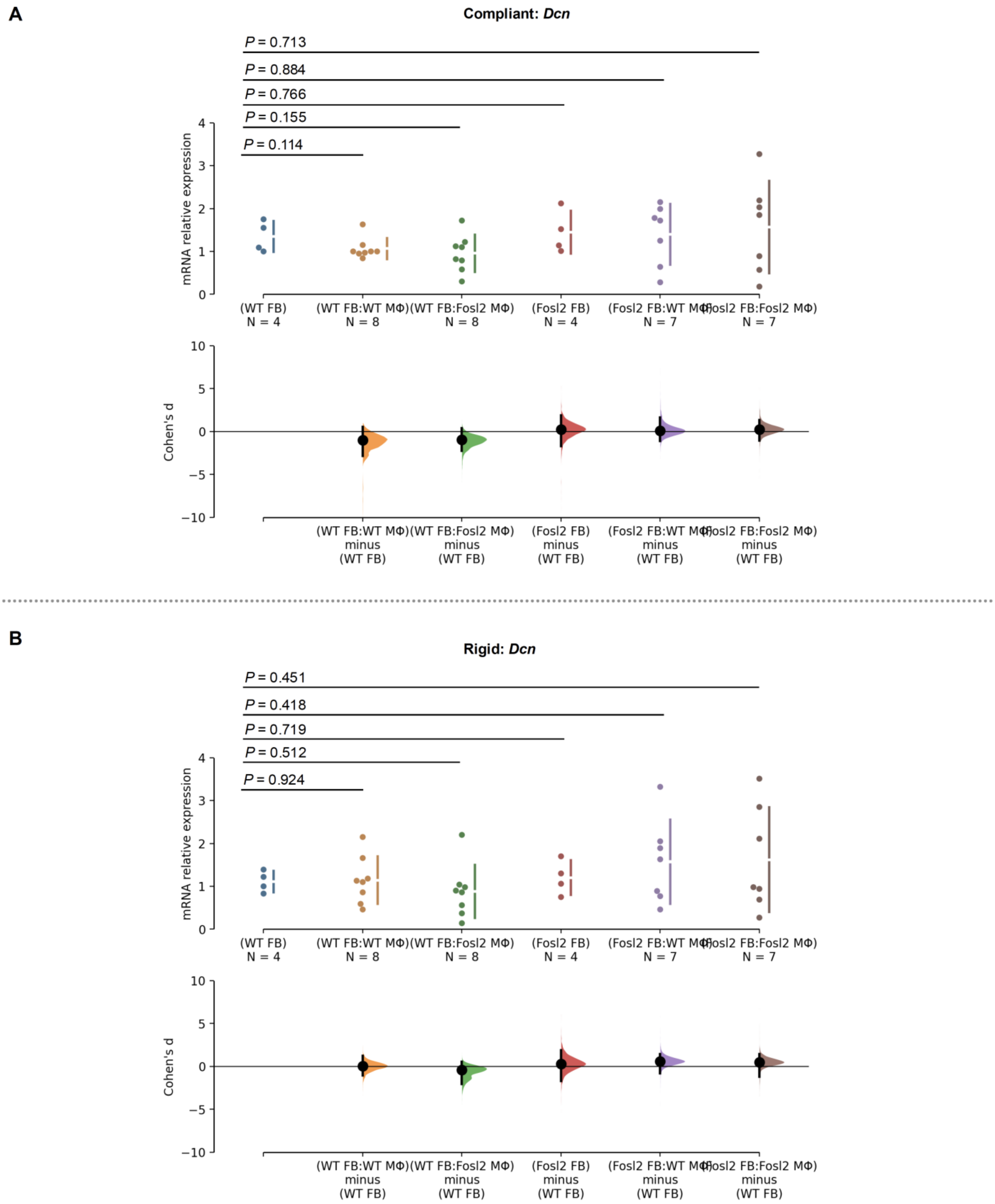
A
Compliant: Dcn
P = 0.713
P = 0.884
P = 0.766
P = 0.155
P = 0.114
mRNA relative expression
(WT FB) N = 4
(WT FB:WT MΦ) N = 8
(WT FB:Fosl2 MΦ) N = 8
(Fosl2 FB) N = 4
(Fosl2 FB:WT MΦ) N = 7
(Fosl2 FB:Fosl2 MΦ) N = 7
Cohen's d
(WT FB:WT MΦ) minus (WT FB)
(WT FB:Fosl2 MΦ) minus (WT FB)
(Fosl2 FB) minus (WT FB)
(Fosl2 FB:WT MΦ) minus (WT FB)
(Fosl2 FB:Fosl2 MΦ) minus (WT FB)
B
Rigid: Dcn
P = 0.451
P = 0.418
P = 0.719
P = 0.512
P = 0.924
mRNA relative expression
(WT FB) N = 4
(WT FB:WT MΦ) N = 8
(WT FB:Fosl2 MΦ) N = 8
(Fosl2 FB) N = 4
(Fosl2 FB:WT MΦ) N = 7
(Fosl2 FB:Fosl2 MΦ) N = 7
Cohen's d
(WT FB:WT MΦ) minus (WT FB)
(WT FB:Fosl2 MΦ) minus (WT FB)
(Fosl2 FB) minus (WT FB)
(Fosl2 FB:WT MΦ) minus (WT FB)
(Fosl2 FB:Fosl2 MΦ) minus (WT FB)

Supplementary Fig. 22 | *Dcn* Gardner–Altman plots for the estimation statistics for data in Figure 7D.
mRNA expression of *Dcn* gene of Fosl-2$^{(Tg)}$ and WT stromal cells in monoculture and direct cocultures tethered to different mechanical boundary rigidities. **(A)** Compliant *Dcn*: The unpaired Cohen's d between (WT FB) and (WT FB:WT MΦ) is -0.989 [95.0%CI -2.83, 0.53]. The unpaired Cohen's d between (WT FB) and (WT FB:Fosl2 MΦ) is -0.946 [95.0%CI -2.27, 0.374]. The unpaired Cohen's d between (WT FB) and (Fosl2 FB) is 0.23 [95.0%CI -1.72, 1.89]. The unpaired Cohen's d between (WT FB) and (Fosl2 FB:WT MΦ) is 0.0877 [95.0%CI -1.09, 1.66]. The unpaired Cohen's d between (WT FB) and (Fosl2 FB:Fosl2 MΦ) is 0.245 [95.0%CI -1.08, 1.32]. **(B)** Rigid *Dcn*: The unpaired Cohen's d between (WT FB) and (WT FB:WT MΦ) is 0.0649 [95.0%CI -1.06, 1.23]. The unpaired Cohen's d between (WT FB) and (WT FB:Fosl2 MΦ) is -0.427 [95.0%CI -2.04, 0.556]. The unpaired Cohen's d between (WT FB) and (Fosl2 FB) is 0.278 [95.0%CI -1.7, 1.9]. The unpaired Cohen's d between (WT FB) and (Fosl2 FB:WT MΦ) is 0.571 [95.0%CI -0.795, 1.45]. The unpaired Cohen's d between (WT FB) and (Fosl2 FB:Fosl2 MΦ) is 0.51 [95.0%CI -1.21, 1.44].

The difference axis of the estimation plot displays the effect size, here the Cohen's *d*. The effect sizes and CIs are reported above as: effect size [CI width lower bound; upper bound]. The 95% confidence interval of Cohen's *d* is illustrated by the black vertical line. The curve displays the distribution of 5000 bootstrap re-samplings. *P* value denotes the two-sided permutation.

36

A

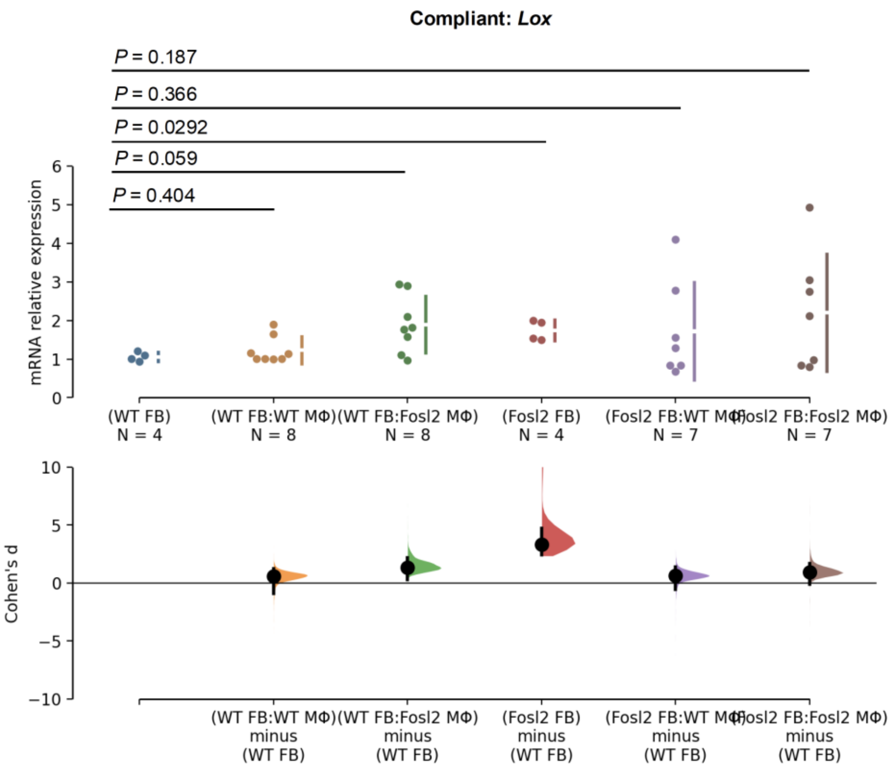
Compliant: Lox
P = 0.187
P = 0.366
P = 0.0292
P = 0.059
P = 0.404
mRNA relative expression
(WT FB)
N = 4
(WT FB:WT MΦ)
N = 8
(WT FB:Fosl2 MΦ)
N = 8
(Fosl2 FB)
N = 4
(Fosl2 FB:WT MΦ)
N = 7
(Fosl2 FB:Fosl2 MΦ)
N = 7
Cohen's d
(WT FB:WT MΦ)
minus
(WT FB)
(WT FB:Fosl2 MΦ)
minus
(WT FB)
(Fosl2 FB)
minus
(WT FB)
(Fosl2 FB:WT MΦ)
minus
(WT FB)
(Fosl2 FB:Fosl2 MΦ)
minus
(WT FB)

B

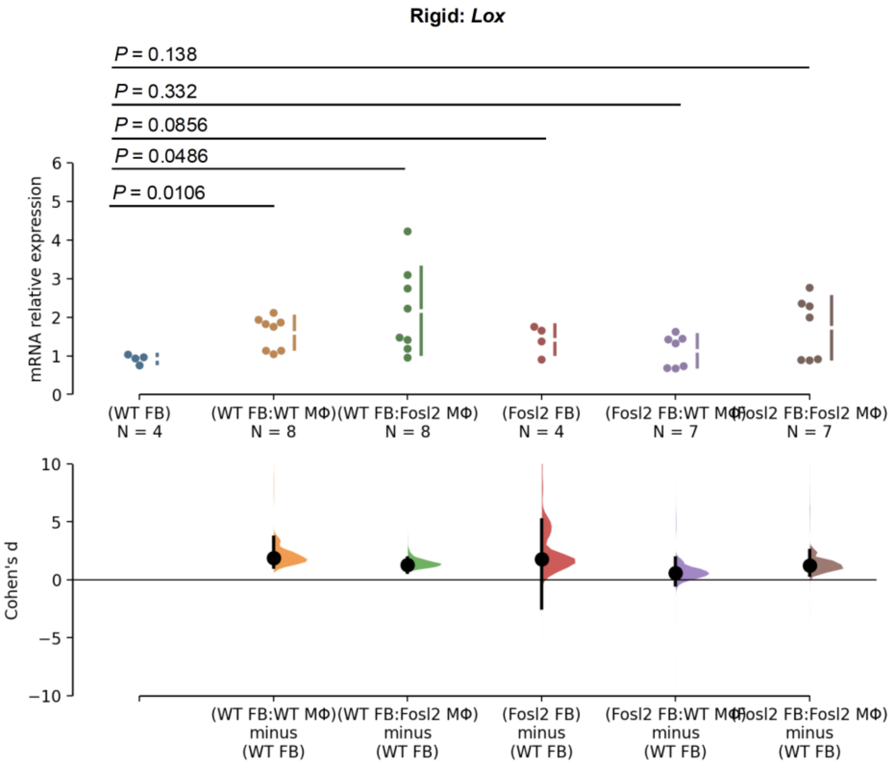
Rigid: Lox
P = 0.138
P = 0.332
P = 0.0856
P = 0.0486
P = 0.0106
mRNA relative expression
(WT FB)
N = 4
(WT FB:WT MΦ)
N = 8
(WT FB:Fosl2 MΦ)
N = 8
(Fosl2 FB)
N = 4
(Fosl2 FB:WT MΦ)
N = 7
(Fosl2 FB:Fosl2 MΦ)
N = 7
Cohen's d
(WT FB:WT MΦ)
minus
(WT FB)
(WT FB:Fosl2 MΦ)
minus
(WT FB)
(Fosl2 FB)
minus
(WT FB)
(Fosl2 FB:WT MΦ)
minus
(WT FB)
(Fosl2 FB:Fosl2 MΦ)
minus
(WT FB)

Supplementary Fig. 23 | *Lox* Gardner–Altman plots for the estimation statistics for data in Figure 7E.
mRNA expression of *Lox* gene of Fosl-2$^{(Tg)}$ and WT stromal cells in monoculture and direct cocultures tethered to different mechanical boundary rigidities. **(A)** Compliant *Lox*: The unpaired Cohen's d between (WT FB) and (WT FB:WT MΦ) is 0.574 [95.0%CI -0.924, 1.25]. The unpaired Cohen's d between (WT FB) and (WT FB:Fosl2 MΦ) is 1.36 [95.0%CI 0.285, 2.16]. The unpaired Cohen's d between (WT FB) and (Fosl2 FB) is 3.34 [95.0%CI 2.44, 4.73]. The unpaired Cohen's d between (WT FB) and (Fosl2 FB:WT MΦ) is 0.639 [95.0%CI -0.55, 1.39]. The unpaired Cohen's d between (WT FB) and (Fosl2 FB:Fosl2 MΦ) is 0.924 [95.0%CI -0.0936, 1.7]. **(B)** Rigid *Lox*: The unpaired Cohen's d between (WT FB) and (WT FB:WT MΦ) is 1.88 [95.0%CI 1.08, 3.7]. The unpaired Cohen's d between (WT FB) and (WT FB:Fosl2 MΦ) is 1.31 [95.0%CI 0.661, 1.89]. The unpaired Cohen's d between (WT FB) and (Fosl2 FB) is 1.77 [95.0%CI -2.45, 5.16]. The unpaired Cohen's d between (WT FB) and (Fosl2 FB:WT MΦ) is 0.603 [95.0%CI -0.442, 1.87]. The unpaired Cohen's d between (WT FB) and (Fosl2 FB:Fosl2 MΦ) is 1.21 [95.0%CI 0.371, 2.53].

The difference axis of the estimation plot displays the effect size, here the Cohen's *d*. The effect sizes and CIs are reported above as: effect size [CI width lower bound; upper bound]. The 95% confidence interval of Cohen's *d* is illustrated by the black vertical line. The curve displays the distribution of 5000 bootstrap re-samplings. *P* value denotes the two-sided permutation.

38

A

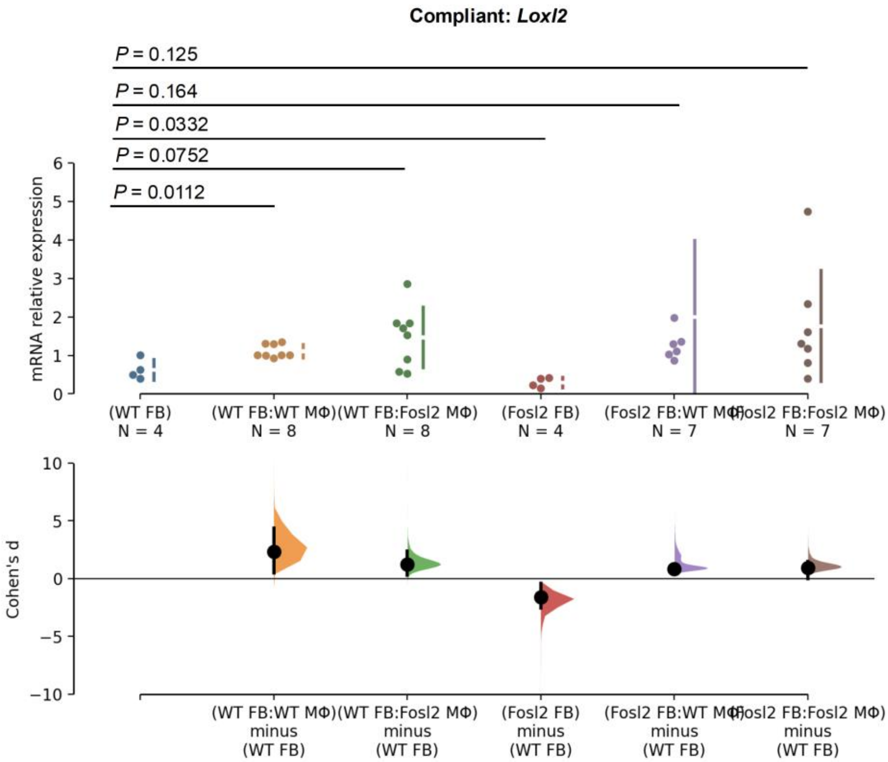
Compliant: Loxl2
P = 0.125
P = 0.164
P = 0.0332
P = 0.0752
P = 0.0112
mRNA relative expression
(WT FB) N = 4
(WT FB:WT MΦ) N = 8
(WT FB:Fosl2 MΦ) N = 8
(Fosl2 FB) N = 4
(Fosl2 FB:WT MΦ) N = 7
(Fosl2 FB:Fosl2 MΦ) N = 7
Cohen's d
(WT FB:WT MΦ) minus (WT FB)
(WT FB:Fosl2 MΦ) minus (WT FB)
(Fosl2 FB) minus (WT FB)
(Fosl2 FB:WT MΦ) minus (WT FB)
(Fosl2 FB:Fosl2 MΦ) minus (WT FB)

B

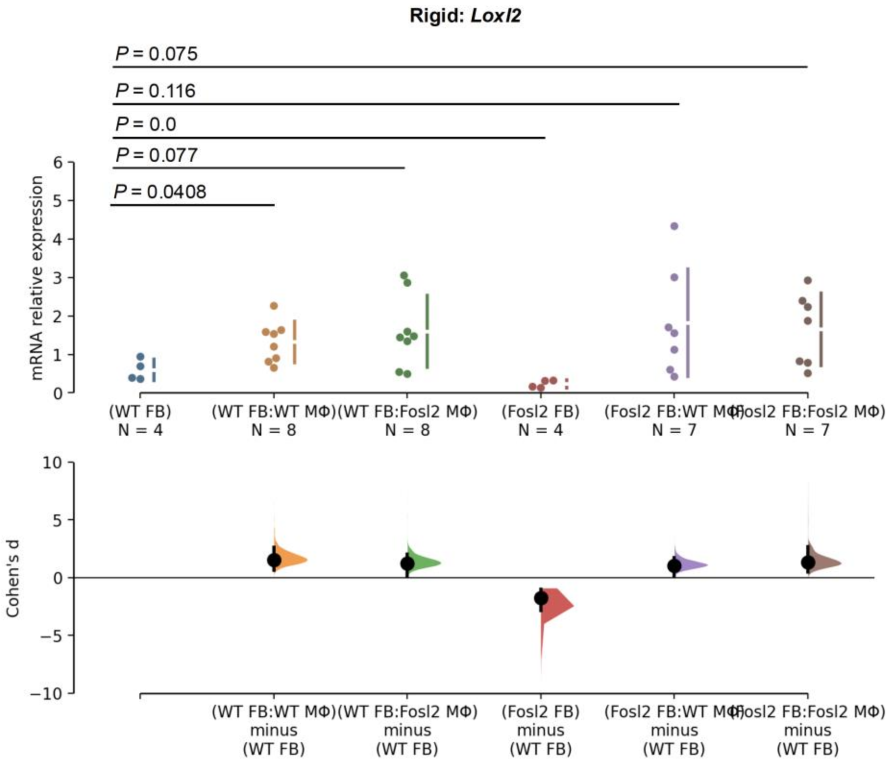
Rigid: Loxl2
P = 0.075
P = 0.116
P = 0.0
P = 0.077
P = 0.0408
mRNA relative expression
(WT FB) N = 4
(WT FB:WT MΦ) N = 8
(WT FB:Fosl2 MΦ) N = 8
(Fosl2 FB) N = 4
(Fosl2 FB:WT MΦ) N = 7
(Fosl2 FB:Fosl2 MΦ) N = 7
Cohen's d
(WT FB:WT MΦ) minus (WT FB)
(WT FB:Fosl2 MΦ) minus (WT FB)
(Fosl2 FB) minus (WT FB)
(Fosl2 FB:WT MΦ) minus (WT FB)
(Fosl2 FB:Fosl2 MΦ) minus (WT FB)

40

Supplementary Fig. 24 | *Loxl2* Gardner–Altman plots for the estimation statistics for data in Figure 7E.
mRNA expression of *Loxl2* gene of Fosl-2$^{(Tg)}$ and WT stromal cells in monoculture and direct cocultures tethered to different mechanical boundary rigidities. **(A)** Compliant *Loxl2*: The unpaired Cohen's d between (WT FB) and (WT FB:WT MΦ) is 2.34 [95.0%CI 0.511, 4.4]. The unpaired Cohen's d between (WT FB) and (WT FB:Fosl2 MΦ) is 1.25 [95.0%CI 0.271, 2.37]. The unpaired Cohen's d between (WT FB) and (Fosl2 FB) is -1.59 [95.0%CI -2.55, -0.409]. The unpaired Cohen's d between (WT FB) and (Fosl2 FB:WT MΦ) is 0.848 [95.0%CI 0.507, 1.12]. The unpaired Cohen's d between (WT FB) and (Fosl2 FB:Fosl2 MΦ) is 0.954 [95.0%CI 0.0165, 1.51]. **(B)** Rigid *Loxl2*: The unpaired Cohen's d between (WT FB) and (WT FB:WT MΦ) is 1.54 [95.0%CI 0.638, 2.66]. The unpaired Cohen's d between (WT FB) and (WT FB:Fosl2 MΦ) is 1.25 [95.0%CI 0.163, 2.04]. The unpaired Cohen's d between (WT FB) and (Fosl2 FB) is -1.77 [95.0%CI -2.86, -0.991]. The unpaired Cohen's d between (WT FB) and (Fosl2 FB:WT MΦ) is 1.06 [95.0%CI 0.165, 1.74]. The unpaired Cohen's d between (WT FB) and (Fosl2 FB:Fosl2 MΦ) is 1.34 [95.0%CI 0.47, 2.7].

The difference axis of the estimation plot displays the effect size, here the Cohen's *d*. The effect sizes and CIs are reported above as: effect size [CI width lower bound; upper bound]. The 95% confidence interval of Cohen's *d* is illustrated by the black vertical line. The curve displays the distribution of 5000 bootstrap re-samplings. *P* value denotes the two-sided permutation.

A

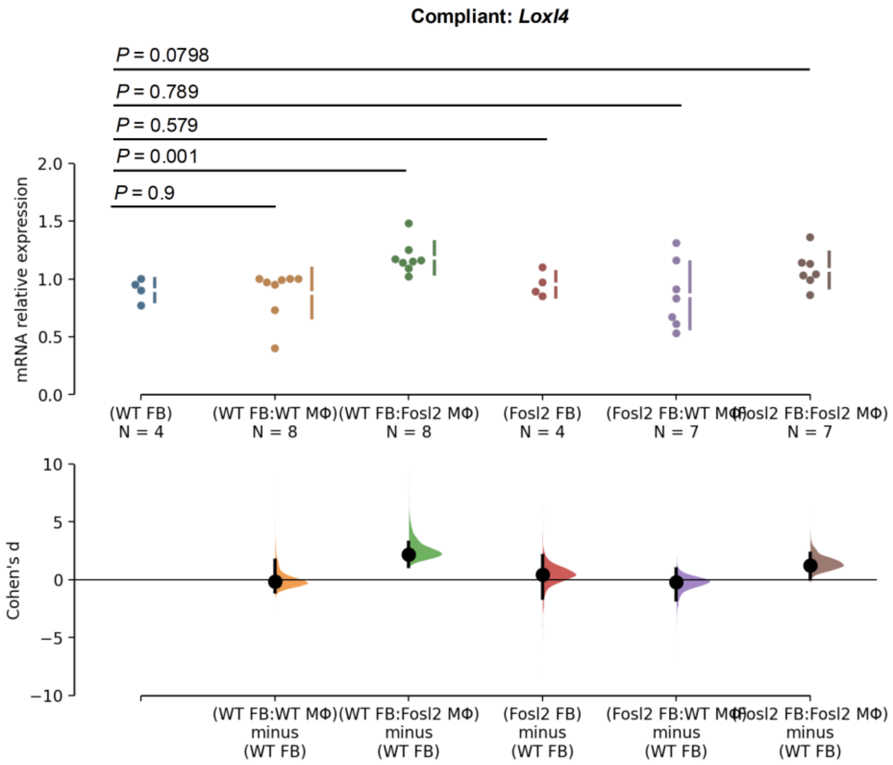
Compliant: *Loxl4*
P = 0.0798
P = 0.789
P = 0.579
P = 0.001
P = 0.9
mRNA relative expression
2.0
1.5
1.0
0.5
0.0
(WT FB)
N = 4
(WT FB:WT MΦ)
N = 8
(WT FB:Fosl2 MΦ)
N = 8
(Fosl2 FB)
N = 4
(Fosl2 FB:WT MΦ)
N = 7
(Fosl2 FB:Fosl2 MΦ)
N = 7
Cohen's d
10
5
0
−5
−10
(WT FB:WT MΦ) minus (WT FB)
(WT FB:Fosl2 MΦ) minus (WT FB)
(Fosl2 FB) minus (WT FB)
(Fosl2 FB:WT MΦ) minus (WT FB)
(Fosl2 FB:Fosl2 MΦ) minus (WT FB)

B

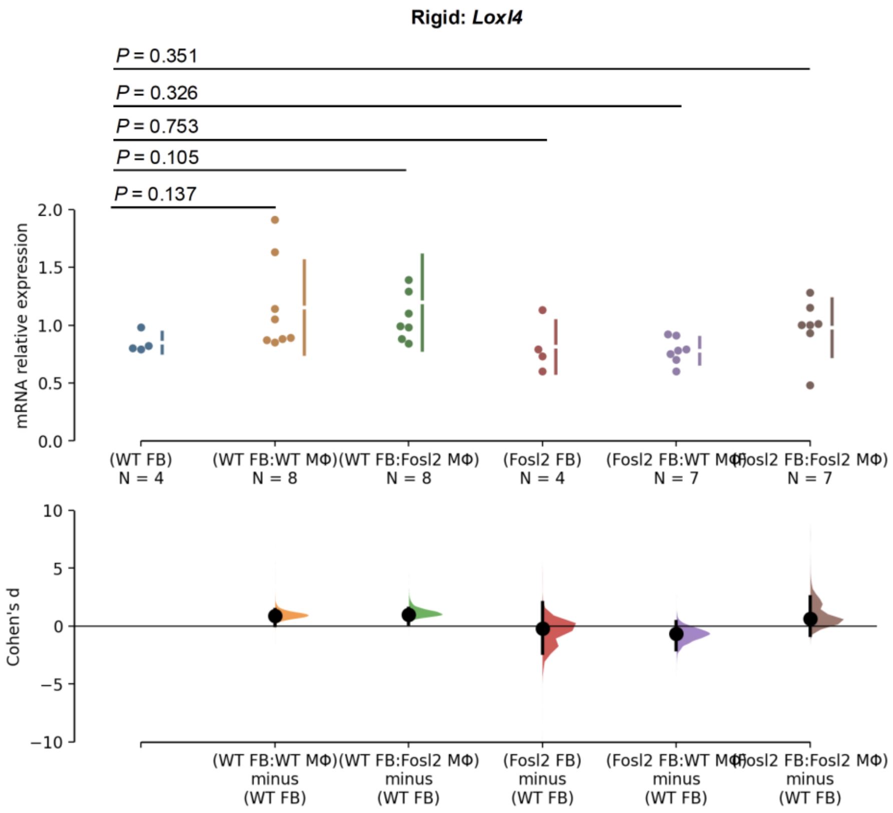
Rigid: *Loxl4*
P = 0.351
P = 0.326
P = 0.753
P = 0.105
P = 0.137
mRNA relative expression
2.0
1.5
1.0
0.5
0.0
(WT FB)
N = 4
(WT FB:WT MΦ)
N = 8
(WT FB:Fosl2 MΦ)
N = 8
(Fosl2 FB)
N = 4
(Fosl2 FB:WT MΦ)
N = 7
(Fosl2 FB:Fosl2 MΦ)
N = 7
Cohen's d
10
5
0
−5
−10
(WT FB:WT MΦ) minus (WT FB)
(WT FB:Fosl2 MΦ) minus (WT FB)
(Fosl2 FB) minus (WT FB)
(Fosl2 FB:WT MΦ) minus (WT FB)
(Fosl2 FB:Fosl2 MΦ) minus (WT FB)

Supplementary Fig. 25 | *Loxl4* Gardner–Altman plots for the estimation statistics for data in Figure 7E.
mRNA expression of *Loxl4* gene of Fosl-2$^{(Tg)}$ and WT stromal cells in monoculture and direct cocultures tethered to different mechanical boundary rigidities.
(A) Compliant *Loxl4*: The unpaired Cohen's d between (WT FB) and (WT FB:WT MΦ) is -0.134 [95.0%CI -1.03, 1.7]. The unpaired Cohen's d between (WT FB) and (WT FB:Fosl2 MΦ) is 2.19 [95.0%CI 1.13, 3.24]. The unpaired Cohen's d between (WT FB) and (Fosl2 FB) is 0.454 [95.0%CI -1.59, 2.1]. The unpaired Cohen's d between (WT FB) and (Fosl2 FB:WT MΦ) is -0.185 [95.0%CI -1.73, 0.956]. The unpaired Cohen's d between (WT FB) and (Fosl2 FB:Fosl2 MΦ) is 1.25 [95.0%CI 0.0432, 2.31]. **(B)** Rigid *Loxl4*: The unpaired Cohen's d between (WT FB) and (WT FB:WT MΦ) is 0.899 [95.0%CI 0.0381, 1.45]. The unpaired Cohen's d between (WT FB) and (WT FB:Fosl2 MΦ) is 1.01 [95.0%CI 0.188, 1.55]. The unpaired Cohen's d between (WT FB) and (Fosl2 FB) is -0.204 [95.0%CI -2.34, 2.03]. The unpaired Cohen's d between (WT FB) and (Fosl2 FB:WT MΦ) is -0.653 [95.0%CI -2.07, 0.401]. The unpaired Cohen's d between (WT FB) and (Fosl2 FB:Fosl2 MΦ) is 0.624 [95.0%CI -0.809, 2.52].

The difference axis of the estimation plot displays the effect size, here the Cohen’s *d*. The effect sizes and CIs are reported above as: effect size [CI width lower bound; upper bound]. The 95% confidence interval of Cohen’s *d* is illustrated by the black vertical line. The curve displays the distribution of 5000 bootstrap re-samplings. *P* value denotes the two-sided permutation.

A

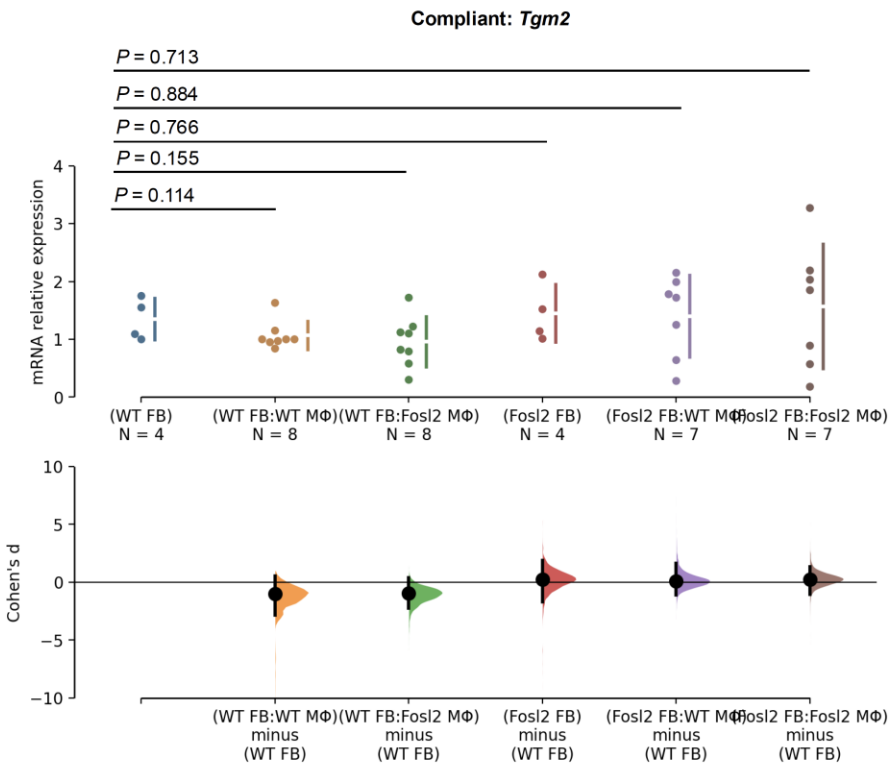

Compliant: Tgm2
P = 0.713
P = 0.884
P = 0.766
P = 0.155
P = 0.114
mRNA relative expression
(WT FB) N = 4
(WT FB:WT MΦ) N = 8
(WT FB:Fosl2 MΦ) N = 8
(Fosl2 FB) N = 4
(Fosl2 FB:WT MΦ) N = 7
(Fosl2 FB:Fosl2 MΦ) N = 7
Cohen's d
(WT FB:WT MΦ) minus (WT FB)
(WT FB:Fosl2 MΦ) minus (WT FB)
(Fosl2 FB) minus (WT FB)
(Fosl2 FB:WT MΦ) minus (WT FB)
(Fosl2 FB:Fosl2 MΦ) minus (WT FB)

B

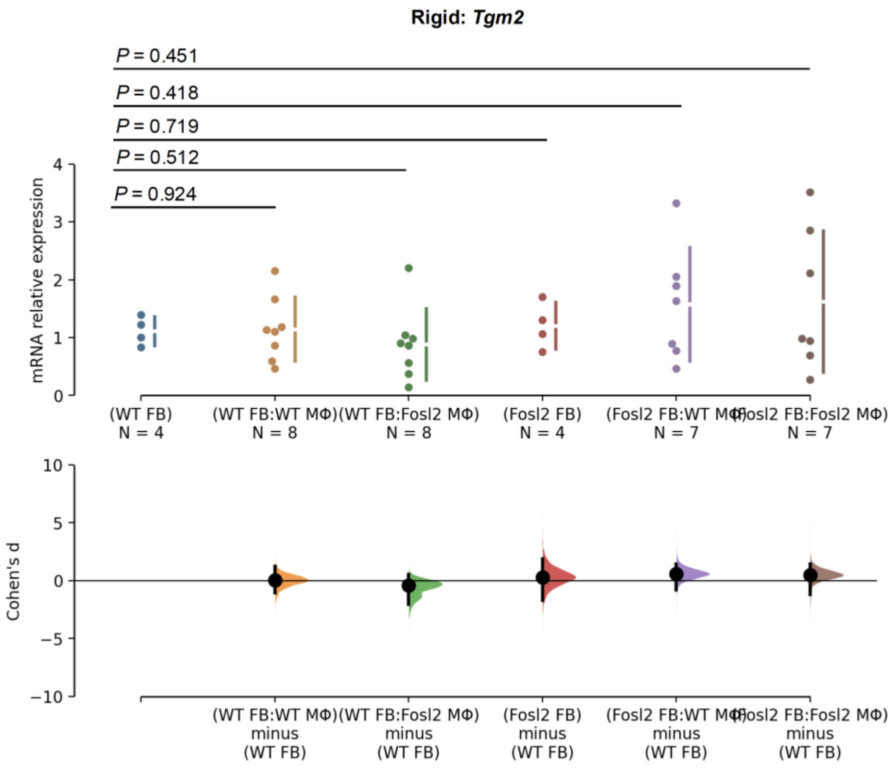

Rigid: Tgm2
P = 0.451
P = 0.418
P = 0.719
P = 0.512
P = 0.924
mRNA relative expression
(WT FB) N = 4
(WT FB:WT MΦ) N = 8
(WT FB:Fosl2 MΦ) N = 8
(Fosl2 FB) N = 4
(Fosl2 FB:WT MΦ) N = 7
(Fosl2 FB:Fosl2 MΦ) N = 7
Cohen's d
(WT FB:WT MΦ) minus (WT FB)
(WT FB:Fosl2 MΦ) minus (WT FB)
(Fosl2 FB) minus (WT FB)
(Fosl2 FB:WT MΦ) minus (WT FB)
(Fosl2 FB:Fosl2 MΦ) minus (WT FB)

Supplementary Fig. 26 | *Tgm2* Gardner–Altman plots for the estimation statistics for data in Figure 7E.
mRNA expression of *Tgm2* gene of Fosl-2$^{(Tg)}$ and WT stromal cells in monoculture and direct cocultures tethered to different mechanical boundary rigidities. **(A)** Compliant *Tgm2*: The unpaired Cohen's d between (WT FB) and (WT FB:WT MΦ) is -0.989 [95.0%CI -2.83, 0.53]. The unpaired Cohen's d between (WT FB) and (WT FB:Fosl2 MΦ) is -0.946 [95.0%CI -2.27, 0.374]. The unpaired Cohen's d between (WT FB) and (Fosl2 FB) is 0.23 [95.0%CI -1.72, 1.89]. The unpaired Cohen's d between (WT FB) and (Fosl2 FB:WT MΦ) is 0.0877 [95.0%CI -1.09, 1.66]. The unpaired Cohen's d between (WT FB) and (Fosl2 FB:Fosl2 MΦ) is 0.245 [95.0%CI -1.08, 1.32]. **(B)** Rigid *Tgm2*: The unpaired Cohen's d between (WT FB) and (WT FB:WT MΦ) is 0.0649 [95.0%CI -1.06, 1.23]. The unpaired Cohen's d between (WT FB) and (WT FB:Fosl2 MΦ) is -0.427 [95.0%CI -2.04, 0.556]. The unpaired Cohen's d between (WT FB) and (Fosl2 FB) is 0.278 [95.0%CI -1.7, 1.9]. The unpaired Cohen's d between (WT FB) and (Fosl2 FB:WT MΦ) is 0.571 [95.0%CI -0.795, 1.45]. The unpaired Cohen's d between (WT FB) and (Fosl2 FB:Fosl2 MΦ) is 0.51 [95.0%CI -1.21, 1.44].

The difference axis of the estimation plot displays the effect size, here the Cohen's *d*. The effect sizes and CIs are reported above as: effect size [CI width lower bound; upper bound]. The 95% confidence interval of Cohen's *d* is illustrated by the black vertical line. The curve displays the distribution of 5000 bootstrap re-samplings. *P* value denotes the two-sided permutation.

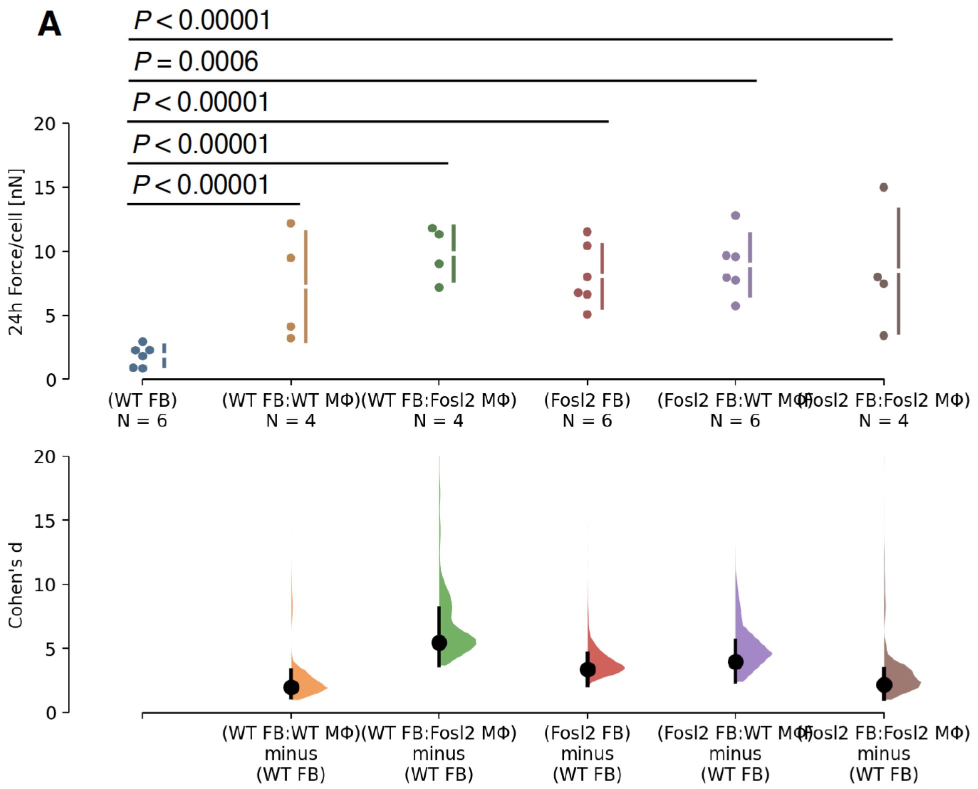

Supplementary Fig. 27 | Gardner–Altman plots for the estimation statistics for data in Figure 7G.
The difference axis of the estimation plot displays the effect size, here the Cohen's *d*. The effect sizes and CIs are reported above as: effect size [CI width lower bound; upper bound]. The 95% confidence interval of Cohen's *d* is illustrated by the black vertical line. The curve displays the distribution of 5000 bootstrap re-samplings. *P* value denotes the two-sided permutation. **(A)** Quantification of tissue traction forces per cell, following normalization to initial seeding density of stromal fibroblasts. *n* = 4-6 tissues from 2-4 mice/genotype. The unpaired Cohen's d between (WT FB) and (WT FB:WT Mϕ) is 1.99 [95.0%CI 1.16, 3.34]. The unpaired Cohen's d between (WT FB) and (WT FB:Fosl2 Mϕ) is 5.44 [95.0%CI 3.68, 8.14]. The unpaired Cohen's d between (WT FB) and (Fosl 2 FB) is 3.39 [95.0%CI 2.13, 4.59]. The unpaired Cohen's d between (WT FB) and (Fosl2 FB:WT Mϕ) is 3.95 [95.0%CI 2.39, 5.62]. The unpaired Cohen's d between (WT FB) and (Fosl2 FB:Fosl2 Mϕ) is 2.2 [95.0%CI 1.05, 3.44].

Supplementary Discussion (Technical *vs.* biological replication):

For all experiments involving the molecular characterization of Fosl-2$^{(Tg)}$ mice, i.e. gene expression and cross-links analysis, we defined single animals as the individual biological unit for replication.

When it comes to the "mechanical" experiments - i.e. the mechano-culture of hydrogels under tension - we find the simplistic intuition behind the conventional dichotomy of "biological" vs. "technical" replication breaks down for multiple reasons. Individual gels compact and develop tension in a way that highly varies between gels, even when cells were originating from the same biological donor (whether human donors or rodents). Our experience consistently showed that the inter-gel/inter-fascicle variability is always higher than the inter-donor/inter-animal variability.[(1)]

To overcome this conceptual challenge, we followed the guiding statistical principles of Lazic *et. al.* who argued that "*the frequent and common distinction made between 'biological' and 'technical' replication is unhelpful because they are inconsistently defined, do not capture the important characteristics of an experiment, and do not clarify what to replicate.*"[(2, 3)] Lazic attributes this shortcoming to oversimplification of the *'biological'* vs. *'technical'* dichotomy which fails to capture the complex, multi-level hierarchy of biological organization. They further argued that it is often that the hypothesis being tested, the experimental interventions, and the technical measurements may each operate at distinct levels of biological organization, thus creating ambiguity regarding which level should dictate the sample size for replication. In an ideal scenario, the hypothesis, interventions, and readouts would all align at the same level of biological organization. However, more often than not, this is not the case as it is the case with our mechano-culture experiments.

Lazic and colleagues propose a hierarchical organization of experimental design differentiating between biological units (BU), experimental units (EU), and observational units (OU).[(3)] Understanding the distinction between these units clarifies where replication should take place. They recommend there are three criteria that must be fulfilled for an experimental unit to qualify as a genuine replicate: 1) Experimental Units (EUs) must be allocated independently to each experimental condition or intervention. 2) Each experimental intervention must be applied independently to every EU. 3) EUs should not exert influence on each other, particularly concerning the measured variable.

Against the backdrop of this recommendation, we defined the individual gels or fascicles as the biological units (BU) and experimental (EU) for replication upon which we aim to test the hypothesis, and the boundary rigidity as the experimental intervention. This way the genuine replication at the level of individual gels (rather than the animal/human donors) is more relevant to the hypothesis we are testing which is about differences in the emergent stiffness of tensioned hydrogels as a function of the boundary rigidity. That is to say, we replicated and perturbed the individual gels and boundary rigidity as a biological unit–intervention pair as the basis for the statistical inference. We believe that this reasoning better captures the nuance related to testing our experimental hypothesis. Furthermore, we are aware of the importance of increasing confidence in any biological findings by testing cells originating from multiple donors/cell lines. As such, we performed all experiments with cell from at least 3 different donors with the statistical inference performed at the level of individual gels rather than the average of gels (from a single donor).

Supplementary Table 1: Patient donor details and demographics

Tissue banking ID	Sex	Tendon	Clinical info
Healthy donors (Age range: 25 – 30)			
772	F	Semitendinosus	ACL autograft tendon transfer, Non-diabetic
819	M	Semitendinosus	ACL autograft tendon transfer, Non-diabetic
933	M	Semitendinosus	ACL autograft tendon transfer, Non-diabetic
Diseased controls (Age range: 55 – 80)			
1250	F	Long head of biceps	Non-diabetic
1273	F	Long head of biceps	Non-diabetic
1275	F	Long head of biceps	Non-diabetic
539	M	Achilles	Non-diabetic
45d	F	Achilles	Non-diabetic
725a	M	Achilles	Diabetic
Systemic sclerosis (Age range: 75 – 80)			
--	F	Biceps<br>Achilles<br>Semitendinosus,<br>Supraspinatus,<br>Infraspinatus	Systemic sclerosis

Supplementary Table 2: RT-qPCR TaqMan primers (Manufacturer: Thermo Fisher Scientific)

Gene	Gene Description	Species	Assay ID	Amplicon Length
Col1a1	collagen, type I, alpha 1	Rat	Rn01463848_m1	115
Col3a1	collagen, type III, alpha 1	Rat	Rn01437681_m1	71
Fn1	fibronectin 1	Rat	Rn00569575_m1	97
Dcn	decorin	Rat	Rn01503161_m1	101
Tnmd	tenomodulin	Rat	Rn00574164_m1	61
Scx	scleraxis	Rat	Rn01504576_m1	63
Mkx	mohawk homeobox	Rat	Rn01755203_m1	60
Acta2	actin, alpha 2, smooth muscle, aorta	Rat	Rn01759928_g1	65
Lox	lysyl oxidase	Rat	Rn01491829_m1	98
Loxl2	lysyl oxidase-like 2	Rat	Rn01466080_m1	76
Loxl4	lysyl oxidase-like 4	Rat	Rn01410872_m1	58
Tgm2	transglutaminase 2	Rat	Rn00571440_m1	67
Eif4a2	eukaryotic translation initiation factor 4A2	Rat	Rn01404749_g1	75
Mmp13	matrix metallopeptidase 13	Rat	Rn01448194_m1	65
Mlk1	MKL/megakaryoblastic leukemia 1	Rat	Rn.PT.58.6636373	
COL1A1	collagen type I alpha 1	Human	Hs00164004_m1	66
COL3A1	collagen type III alpha 1 chain	Human	Hs00943809_m1	65
FN1	fibronectin 1	Human	Hs01549976_m1	81
ACTA2	actin, alpha 2, smooth muscle, aorta	Human	Hs00426835_g1	105
TLR4	toll like receptor 4	Human	Hs00152939_m1	89
IL6	interleukin 6	Human	Hs00174131_m1	95
CXCL8 (IL8)	C-X-C motif chemokine ligand 8	Human	Hs00174103_m1	101
CCL2	C-C motif chemokine ligand 2	Human	Hs00234140_m1	102
PDPN	podoplanin	Human	Hs00366766_m1	58
VCAM1	vascular cell adhesion molecule 1	Human	Hs01003372_m1	62
ICAM1	intercellular adhesion molecule 1	Human	Hs00164932_m1	87
LOXL2	lysyl oxidase like 2	Human	Hs00158757_m1	62

Supplementary References

1. Wunderli SL, Widmer J, Amrein N, Foolen J, Silvan U, Leupin O, et al. Minimal mechanical load and tissue culture conditions preserve native cell phenotype and morphology in tendon-a novel ex vivo mouse explant model. J Orthop Res. 2018;36(5):1383-90.

2. Lazic SE. Experimental design for laboratory biologists: maximising information and improving reproducibility: Cambridge University Press; 2016.

3. Lazic SE, Clarke-Williams CJ, Munafò MR. What exactly is *'N'* in cell culture and animal experiments? Plos Biology. 2018;16(4).
