# Supplementary material for: Mechanical gating of tendon fibrogenic transcription in systemic sclerosis
Source: Nat Commun. 2026 Mar 13;17:3893. doi: 10.1038/s41467-026-70395-2 (PMC13125569; doi:10.1038/s41467-026-70395-2)
Supplement: Supplementary file 2 — Reporting Summary [file 41467_2026_70395_MOESM2_ESM.pdf]

nature portfolio

Corresponding author(s): Jess G. Snedeker

Last updated by author(s): Feb 18, 2026

Reporting Summary

Nature Portfolio wishes to improve the reproducibility of the work that we publish. This form provides structure for consistency and transparency in reporting. For further information on Nature Portfolio policies, see our Editorial Policies and the Editorial Policy Checklist.

Statistics

For all statistical analyses, confirm that the following items are present in the figure legend, table legend, main text, or Methods section.

n/a	Confirmed	
☐	☒	The exact sample size (*n*) for each experimental group/condition, given as a discrete number and unit of measurement
☐	☒	A statement on whether measurements were taken from distinct samples or whether the same sample was measured repeatedly
☐	☒	The statistical test(s) used AND whether they are one- or two-sided<br>*Only common tests should be described solely by name; describe more complex techniques in the Methods section.*
☐	☒	A description of all covariates tested
☐	☒	A description of any assumptions or corrections, such as tests of normality and adjustment for multiple comparisons
☐	☒	A full description of the statistical parameters including central tendency (e.g. means) or other basic estimates (e.g. regression coefficient) AND variation (e.g. standard deviation) or associated estimates of uncertainty (e.g. confidence intervals)
☐	☒	For null hypothesis testing, the test statistic (e.g. *F*, *t*, *r*) with confidence intervals, effect sizes, degrees of freedom and *P* value noted<br>*Give P values as exact values whenever suitable.*
☒	☐	For Bayesian analysis, information on the choice of priors and Markov chain Monte Carlo settings
☒	☐	For hierarchical and complex designs, identification of the appropriate level for tests and full reporting of outcomes
☐	☒	Estimates of effect sizes (e.g. Cohen's *d*, Pearson's *r*), indicating how they were calculated

Our web collection on statistics for biologists contains articles on many of the points above.

Software and code

Policy information about availability of computer code

Data collection	TILL Photonics Life Acquisition, Leica TCS SP8, Motic Images Plus 2.0, STARe Excellence Thermal Analysis Software (METTLER TOLEDO), StepOne™ and StepOnePlus™ Software (Applied Biosystem), The FT-W1002 Mechanical Testing and Handling Software Suite (Femto-Tools AG, Switzerland), Gen5 for Microplate Reading & Data Analysis (Biotek).
Data analysis	Matlab® R2018b (MathWorks, Inc.), Ansys Workbench (v. 16.2., ANSYS, Inc), Prism 10.3.0 (GraphPad Software), DABEST-Matlab for estimation statistics, ImageJ 2.0.0 (National Institutes of Health), Microsoft Excel for Mac (v. 16.51), Custom Matlab scripts (Quantification of posts traction forces). Bioinformatics: eVITTA, CIBERSORTx and Metascape packages.<br><br>Custom Matlab code developed in this manuscript is publicly available at Zenodo under accession: 18455078 (https://doi.org/10.5281/zenodo.18455077)

For manuscripts utilizing custom algorithms or software that are central to the research but not yet described in published literature, software must be made available to editors and reviewers. We strongly encourage code deposition in a community repository (e.g. GitHub). See the Nature Portfolio guidelines for submitting code & software for further information.

Data

Policy information about availability of data

All manuscripts must include a data availability statement. This statement should provide the following information, where applicable:

- Accession codes, unique identifiers, or web links for publicly available datasets
- A description of any restrictions on data availability
- For clinical datasets or third party data, please ensure that the statement adheres to our policy

Source data files are provided with this paper. The RNA-sequencing data generated in this study have been deposited in the Gene Expression Omnibus (GEO) database under accession number GSE319654 (https://www.ncbi.nlm.nih.gov/geo/query/acc.cgi?acc=GSE319654). The raw data that support the findings of this study are available from the corresponding author upon request.

Field-specific reporting

Please select the one below that is the best fit for your research. If you are not sure, read the appropriate sections before making your selection.

☒ Life sciences ☐ Behavioural & social sciences ☐ Ecological, evolutionary & environmental sciences

For a reference copy of the document with all sections, see nature.com/documents/nr-reporting-summary-flat.pdf

Life sciences study design

All studies must disclose on these points even when the disclosure is negative.

Sample size	No statistical methods were used to pre-determine sample size. Sample sizes in our study are in the range of of previous work reported by our lab and others: (Foolen J et. al. Matrix Biol. 2018 Jan;65:14-29, Sakar MS et. al. Nat Commun. 2016 Mar 16;7:11036, Kural MH et. al. Biomaterials. 2014 Jan;35(4):1128-37).
Data exclusions	No data were excluded from the analyses. However, We note that variability in replicate numbers (n) across some experiments is due to multiple factors. In mechano-culture experiments, this variability resulted from rupture or degradation of collagen hydrogels during the experiments. In Fosl-2(Tg) experiments, it arose from low RNA yield in Fosl-2 tendons, as tissue crosslinking makes RNA extraction particularly challenging. Additionally, Fosl-2(Tg) mice often develop a severe phenotype that necessitates euthanasia for welfare reasons, leading to a relatively high dropout rate compared with WT controls within each litter. When sample numbers were limited, we prioritized the most important readouts.
Replication	We performed multiple independent experiments, as described in figure legends.
Randomization	The experiments were not randomized. However, materials used for preparing hydrogels were from different batches, force quantification plates were assembled and used in random fashion, and animals were randomly selected from different litters.
Blinding	The investigators were not blinded, as the same investigators who set up the experiments carried out the analyses.

Reporting for specific materials, systems and methods

We require information from authors about some types of materials, experimental systems and methods used in many studies. Here, indicate whether each material, system or method listed is relevant to your study. If you are not sure if a list item applies to your research, read the appropriate section before selecting a response.

Materials & experimental systems

n/a	Involved in the study	
☐	☒	Antibodies
☒	☐	Eukaryotic cell lines
☒	☐	Palaeontology and archaeology
☐	☒	Animals and other organisms
☐	☒	Human research participants
☒	☐	Clinical data
☒	☐	Dual use research of concern

Methods

n/a	Involved in the study	
☒	☐	ChIP-seq
☒	☐	Flow cytometry
☒	☐	MRI-based neuroimaging

Antibodies

Antibodies used	All antibodies used in this study were purchased commercially.<br><br>- Anti-alpha smooth muscle Actin antibody, Abcam (ab5694), dilution: 1:200. Lot# GR283004-12<br>- Donkey anti-Rabbit IgG (H+L) Highly Cross-Adsorbed Secondary Antibody, Alexa Fluor 488, Life Technologies (A21206), dilution:

	1:500. Lot# 1754421
Validation	The used antibodies were previously published and validated by the manufacturer.<br><br>https://www.abcam.com/alpha-smooth-muscle-actin-antibody-ab5694.html

Animals and other organisms

Policy information about studies involving animals; ARRIVE guidelines recommended for reporting animal research

Laboratory animals	Rats: Wistar (from Janvier-Labs), Male. Age: 12-16 week-old.<br><br>Mice:<br>Fosl-2Tg mice in C57Bl6/J background. Both male and female mice were used. Age: 6-24 week-old.<br>All mice were housed in pathogen free conditions at the University of Zurich or ETH Zurich facilities.
Wild animals	No wild animals were used in this study.
Field-collected samples	The study did not involve samples collected from the field.
Ethics oversight	All experiments involving animals or animal-derived cells were approved by the Zurich Cantonal Veterinary Office. (Permits: ZH007/2019 Fosl-2Tg, ZH265/14 - ZH239/17 Wistar rats).

Note that full information on the approval of the study protocol must also be provided in the manuscript.

Human research participants

Policy information about studies involving human research participants

Population characteristics	Healthy tendon stromal cells were obtained from young donors, male and female (Age: 18+25 year-old). Healthy tissue samples were from donors aged (25-30) years old. Disease tissue were from donors aged between 55-80 years old. Systemic sclerosis samples were from a single (Age range 75-80 year-old) female donor with confirmed clinical diagnosis of systemic sclerosis. Detailed characteristics are included in supplementary table S1. Broad age-ranges were provided for each cohort.
Recruitment	Donors were not specifically recruited for this study. Human samples were collected with written informed donor consent with voluntary participation, in compliance with the requirements of Declaration of Helsinki, Swiss Federal Human Research Act (HRA), and Zurich Cantonal Ethics Commission (Approval numbers: 2015-0089, 2020-0119). Excess tendon tissues were collected intra-operatively and only waste tissue that would otherwise have been discarded was collected.<br><br>All donors signed written informed consent in accordance with the requirements of the Declaration of Helsinki, and the Swiss Federal Human Research Act (HRA).
Ethics oversight	Human tissues collection was approved by Zurich Cantonal Ethics Commission. Approval numbers: 2015-684 0089, 2020-0119).

Note that full information on the approval of the study protocol must also be provided in the manuscript.
